# A principal component meta-analysis on multiple anthropometric traits identifies novel loci for body shape

**DOI:** 10.1038/ncomms13357

**Published:** 2016-11-23

**Authors:** Janina S. Ried, Janina Jeff M., Audrey Y. Chu, Jennifer L. Bragg-Gresham, Jenny van Dongen, Jennifer E. Huffman, Tarunveer S. Ahluwalia, Gemma Cadby, Niina Eklund, Joel Eriksson, Tõnu Esko, Mary F. Feitosa, Anuj Goel, Mathias Gorski, Caroline Hayward, Nancy L. Heard-Costa, Anne U. Jackson, Eero Jokinen, Stavroula Kanoni, Kati Kristiansson, Zoltán Kutalik, Jari Lahti, Jian'an Luan, Reedik Mägi, Anubha Mahajan, Massimo Mangino, Carolina Medina-Gomez, Keri L. Monda, Ilja M. Nolte, Louis Pérusse, Inga Prokopenko, Lu Qi, Lynda M. Rose, Erika Salvi, Megan T. Smith, Harold Snieder, Alena Stančáková, Yun Ju Sung, Ioanna Tachmazidou, Alexander Teumer, Gudmar Thorleifsson, Pim van der Harst, Ryan W. Walker, Sophie R. Wang, Sarah H. Wild, Sara M. Willems, Andrew Wong, Weihua Zhang, Eva Albrecht, Alexessander Couto Alves, Stephan J. L. Bakker, Cristina Barlassina, Traci M. Bartz, John Beilby, Claire Bellis, Richard N. Bergman, Sven Bergmann, John Blangero, Matthias Blüher, Eric Boerwinkle, Lori L. Bonnycastle, Stefan R. Bornstein, Marcel Bruinenberg, Harry Campbell, Yii-Der Ida Chen, Charleston W. K. Chiang, Peter S. Chines, Francis S Collins, Fracensco Cucca, L Adrienne Cupples, Francesca D'Avila, Eco J .C. de Geus, George Dedoussis, Maria Dimitriou, Angela Döring, Johan G. Eriksson, Aliki-Eleni Farmaki, Martin Farrall, Teresa Ferreira, Krista Fischer, Nita G. Forouhi, Nele Friedrich, Anette Prior Gjesing, Nicola Glorioso, Mariaelisa Graff, Harald Grallert, Niels Grarup, Jürgen Gräßler, Jagvir Grewal, Anders Hamsten, Marie Neergaard Harder, Catharina A. Hartman, Maija Hassinen, Nicholas Hastie, Andrew Tym Hattersley, Aki S. Havulinna, Markku Heliövaara, Hans Hillege, Albert Hofman, Oddgeir Holmen, Georg Homuth, Jouke-Jan Hottenga, Jennie Hui, Lise Lotte Husemoen, Pirro G. Hysi, Aaron Isaacs, Till Ittermann, Shapour Jalilzadeh, Alan L. James, Torben Jørgensen, Pekka Jousilahti, Antti Jula, Johanne Marie Justesen, Anne E. Justice, Mika Kähönen, Maria Karaleftheri, Kay Tee Khaw, Sirkka M. Keinanen-Kiukaanniemi, Leena Kinnunen, Paul B. Knekt, Heikki A. Koistinen, Ivana Kolcic, Ishminder K. Kooner, Seppo Koskinen, Peter Kovacs, Theodosios Kyriakou, Tomi Laitinen, Claudia Langenberg, Alexandra M. Lewin, Peter Lichtner, Cecilia M. Lindgren, Jaana Lindström, Allan Linneberg, Roberto Lorbeer, Mattias Lorentzon, Robert Luben, Valeriya Lyssenko, Satu Männistö, Paolo Manunta, Irene Mateo Leach, Wendy L. McArdle, Barbara Mcknight, Karen L. Mohlke, Evelin Mihailov, Lili Milani, Rebecca Mills, May E. Montasser, Andrew P. Morris, Gabriele Müller, Arthur W. Musk, Narisu Narisu, Ken K. Ong, Ben A. Oostra, Clive Osmond, Aarno Palotie, James S. Pankow, Lavinia Paternoster, Brenda W. Penninx, Irene Pichler, Maria G. Pilia, Ozren Polašek, Peter P. Pramstaller, Olli T Raitakari, Tuomo Rankinen, D. C. Rao, Nigel W. Rayner, Rasmus Ribel-Madsen, Treva K. Rice, Marcus Richards, Paul M. Ridker, Fernando Rivadeneira, Kathy A. Ryan, Serena Sanna, Mark A. Sarzynski, Salome Scholtens, Robert A. Scott, Sylvain Sebert, Lorraine Southam, Thomas Hempel Sparsø, Valgerdur Steinthorsdottir, Kathleen Stirrups, Ronald P. Stolk, Konstantin Strauch, Heather M. Stringham, Morris A. Swertz, Amy J. Swift, Anke Tönjes, Emmanouil Tsafantakis, Peter J. van der Most, Jana V. Van Vliet-Ostaptchouk, Liesbeth Vandenput, Erkki Vartiainen, Cristina Venturini, Niek Verweij, Jorma S. Viikari, Veronique Vitart, Marie-Claude Vohl, Judith M. Vonk, Gérard Waeber, Elisabeth Widén, Gonneke Willemsen, Tom Wilsgaard, Thomas W. Winkler, Alan F. Wright, Laura M. Yerges-Armstrong, Jing Hua Zhao, M. Carola Zillikens, Dorret I. Boomsma, Claude Bouchard, John C. Chambers, Daniel I. Chasman, Daniele Cusi, Ron T. Gansevoort, Christian Gieger, Torben Hansen, Andrew A. Hicks, Frank Hu, Kristian Hveem, Marjo-Riitta Jarvelin, Eero Kajantie, Jaspal S. Kooner, Diana Kuh, Johanna Kuusisto, Markku Laakso, Timo A. Lakka, Terho Lehtimäki, Andres Metspalu, Inger Njølstad, Claes Ohlsson, Albertine J. Oldehinkel, Lyle J. Palmer, Oluf Pedersen, Markus Perola, Annette Peters, Bruce M. Psaty, Hannu Puolijoki, Rainer Rauramaa, Igor Rudan, Veikko Salomaa, Peter E. H. Schwarz, Alan R. Shudiner, Jan H. Smit, Thorkild I. A. Sørensen, Timothy D. Spector, Kari Stefansson, Michael Stumvoll, Angelo Tremblay, Jaakko Tuomilehto, André G. Uitterlinden, Matti Uusitupa, Uwe Völker, Peter Vollenweider, Nicholas J. Wareham, Hugh Watkins, James F. Wilson, Eleftheria Zeggini, Goncalo R. Abecasis, Michael Boehnke, Ingrid B. Borecki, Panos Deloukas, Cornelia M. van Duijn, Caroline Fox, Leif C. Groop, Iris M. Heid, David J. Hunter, Robert C. Kaplan, Mark I. McCarthy, Kari E. North, Jeffrey R. O'Connell, David Schlessinger, Unnur Thorsteinsdottir, David P. Strachan, Timothy Frayling, Joel N. Hirschhorn, Martina Müller-Nurasyid, Ruth J. F. Loos

**Affiliations:** 1 Institute of Genetic Epidemiology, Helmholtz Zentrum München-German Research Center for Environmental Health, 85764 Neuherberg, Germany; 2 The Charles Bronfman Institute for Personalized Medicine, The Icahn School of Medicine at Mount Sinai, New York, New York 10029, USA; 3 Division of Preventive Medicine, Brigham and Women's Hospital, Boston, Massachusetts 02215, USA; 4 Kidney Epidemiology and Cost Center, Internal Medicine-Nephrology, University of Michigan, Ann Arbor, Michigan 48109, USA; 5 Department of Biological Psychology, VU University, 1081BT Amsterdam, The Netherlands; 6 MRC Human Genetics Unit, Institute of Genetics and Molecular Medicine, University of Edinburgh, EH4 2XU Edinburgh, Scotland; 7 Faculty of Health and Medical Sciences, Novo Nordisk Foundation Center for Basic Metabolic Research, Section of Metabolic Genetics, University of Copenhagen, 2100 Copenhagen, Denmark; 8 Steno Diabetes Center A/S, DK-2820 Gentofte, Denmark; 9 COPSAC, Copenhagen Prospective Studies on Asthma in Childhood, Herlev and Gentofte Hospital, University of Copenhagen, Ledreborg Allé 34, DK-2820 Copenhagen, Denmark; 10 Centre for Genetic Origins of Health and Disease, University of Western Australia, Crawley, Western Australia 6009, Australia; 11 Department of Health, National Institute for Health and Welfare (THL), FI-00271 Helsinki, Finland; 12 Department of Internal Medicine and Clinical Nutrition, Centre for Bone and Arthritis Research, Institute of Medicine, Sahlgrenska Academy, University of Gothenburg, 413 45 Gothenburg, Sweden; 13 Broad Institute of the Massachusetts Institute of Technology and Harvard University, Cambridge, Massachusetts 2142, USA; 14 Divisions of Endocrinology and Genetics and Center for Basic and Translational Obesity Research, Boston Children's Hospital, Boston, Massachusetts 02115, USA; 15 Estonian Genome Center, University of Tartu, Tartu 51010, Estonia; 16 Department of Genetics, Harvard Medical School, Boston, Massachusetts 02115, USA; 17 Division of Statistical Genomics, Department of Genetics, Washington University School of Medicine, St. Louis, Missouri 63108, USA; 18 Division of Cardiovascular Medicine, Radcliffe Department of Medicine, University of Oxford, Oxford OX3 9DU, UK; 19 Wellcome Trust Centre for Human Genetics, University of Oxford, Oxford OX3 7BN, UK; 20 Department of Nephrology, University Hospital Regensburg, 93042 Regensburg, Germany; 21 Department of Genetic Epidemiology, Institute of Epidemiology and Preventive Medicine, University of Regensburg, 93053 Regensburg, Germany; 22 National Heart, Lung, and Blood Institute, the Framingham Heart Study, Framingham, Massachusetts 01702, USA; 23 Department of Neurology, Boston University School of Medicine, Boston, Massachusetts 02118, USA; 24 Department of Biostatistics, Center for Statistical Genetics, University of Michigan, Ann Arbor, Michigan 48109, USA; 25 Hospital for Children and Adolescents, University of Helsinki, FI-00290 Helsinki, Finland; 26 Wellcome Trust Sanger Institute, Human Genetics, Hinxton, Cambridge CB10 1SA, UK; 27 William Harvey Research Institute, Barts and The London School of Medicine and Dentistry, Queen Mary University of London, London EC1M 6BQ, UK; 28 Institute for Molecular Medicine Finland, University of Helsinki, FI-00290 Helsinki, Finland; 29 Swiss Institute of Bioinformatics, 1015 Lausanne, Switzerland; 30 Department of Medical Genetics, University of Lausanne, Lausanne, 1005, Switzerland; 31 Institute of Social and Preventive Medicine, University Hospital Lausanne (CHUV), 1010 Lausanne, Switzerland; 32 Folkhälsan Research Centre, FI-00290 Helsinki, Finland; 33 Institute of Behavioural Sciences, University of Helsinki, FI-00014 Helsinki, Finland; 34 MRC Epidemiology Unit, University of Cambridge School of Clinical Medicine, Institute of Metabolic Science, University of Cambridge, Cambridge Biomedical Campus, Cambridge CB2 0QQ, UK; 35 Department of Twin Research and Genetic Epidemiology, King's College London, London SE1 7EH, UK; 36 Department of Epidemiology, Erasmus Medical Center, 3015GE Rotterdam, The Netherlands; 37 Department of Internal Medicine, Erasmus Medical Center, 3015GE Rotterdam, The Netherlands; 38 Department of Epidemiology, University of North Carolina at Chapel Hill, Chapel Hill, North Carolina 27599, USA; 39 The Center for Observational Research, Amgen Inc., Thousand Oaks, California 91320-1799, USA; 40 Department of Epidemiology, University of Groningen, University Medical Center Groningen, 9700 RB Groningen, The Netherlands; 41 Department of Kinesiology, Laval University, Québec, Québec, Canada G1V 0A6; 42 Institute of Nutrition and Functional Foods, Laval University, Québec, Québec, Canada G1V 0A6; 43 Department of Genomics of Common Disease, School of Public Health, Imperial College London, London W12 0NN, UK; 44 Oxford Centre for Diabetes, Endocrinology and Metabolism, University of Oxford, Churchill Hospital, Oxford OX3 7LJ, UK; 45 Department of Medicine, Channing Division of Network Medicine, Brigham and Women's Hospital and Harvard Medical School, Boston, Massachusetts 02115, USA; 46 Department of Nutrition, Harvard School of Public Health, Boston, Massachusetts 02115, USA; 47 Department of Health Sciences, University of Milano at San Paolo Hospital, 20139 Milano, Italy; 48 Filarete Foundation, Genomic and Bioinformatics Unit, Milano 20139, Italy; 49 Department of Biostatistics, University of Washington, Seattle, Washington 98195, USA; 50 Department of Medicine, University of Eastern Finland and Kuopio University Hospital, 70210 Kuopio, Finland; 51 Division of Biostatistics, Washington University School of Medicine, St. Louis, Missouri 63110, USA; 52 Institute for Community Medicine, University Medicine Greifswald, 17475 Greifswald, Germany; 53 Interfaculty Institute for Genetics and Functional Genomics, University Medicine Greifswald, 17475 Greifswald, Germany; 54 deCODE Genetics, Amgen inc., 101 Reykjavik, Iceland; 55 Durrer Center for Cardiogenetic Research, Interuniversity Cardiology Institute Netherlands-Netherlands Heart Institute, 3501 DG Utrecht, The Netherlands; 56 Department of Genetics, University of Groningen, University Medical Center Groningen, 9700 RB Groningen, The Netherlands; 57 Department of Cardiology, University of Groningen, University Medical Center Groningen, 9700 RB Groningen, Netherlands; 58 The Department of Preventive Medicine, The Icahn School of Medicine at Mount Sinai, New York, New York 10029, USA; 59 Program in Medical and Population Genetics, Broad Institute of Harvard and Massachusetts Institute of Technology, Cambridge, Massachusetts 02142, USA; 60 Division of Endocrinology, Boston Children's Hospital, Boston, Massachusetts 02115, USA; 61 Divisions of Genetics and Endocrinology and Program in Genomics, Boston's Children's Hospital, Boston, Massachusetts 02115, USA; 62 Centre for Global Health Research, Usher Institute of Population Health Sciences and Informatics, University of Edinburgh, EH8 9AG Teviot Place, Edinburgh, Scotland; 63 Department of Epidemiology, Genetic Epidemiology Unit, Erasmus University Medical Center, 3015GE Rotterdam, The Netherlands; 64 MRC Unit for Lifelong Health & Ageing at UCL, London WC1B 5JU, UK; 65 Department of Epidemiology and Biostatistics, Imperial College London, London W2 1PG, UK; 66 Ealing Hospital NHS Trust, Middlesex UB1 3HW, UK; 67 Department of Epidemiology and Biostatistics, MRC Health Protection Agency (HPA) Centre for Environment and Health, School of Public Health, Imperial College, London W12 0NN, UK; 68 Department of Medicine, University of Groningen, University Medical Center Groningen, 9700 RB Groningen, Netherlands; 69 Department of Medicine, University of Washington, Seattle, Washington 98101, USA; 70 Cardiovascular Health Research Unit, University of Washington, Seattle, Washington 98101, USA; 71 Pathwest Laboratory Medicine of Western Australia, Nedlands, Western Australia 6009, Australia; 72 School of Pathology and Laboratory Medicine, University of Western Australia, Nedlands, Western Australia 6009, Australia; 73 Genomics Research Centre, Institute of Health and Biomedical Innovation, Queensland University of Technology, Brisbane, Queensland 4001, Australia; 74 Human Genetics, Genome Institute of Singapore, Agency for Science, Technology and Research of Singapore, Singapore 138672, Singapore; 75 Diabetes and Obesity Research Institute, Cedars-Sinai Medical Center, Los Angeles, California 90048, USA; 76 South Texas Diabetes and Obesity Institute, University of Texas Rio Grande Valley, Brownsville, Texas 78520, USA; 77 University of Leipzig, IFB Adiposity Diseases, 04103 Leipzig, Germany; 78 Department of Medicine, University of Leipzig, 04103 Leipzig, Germany; 79 Human Genetics Center and Institute of Molecular Medicine, University of Texas Health Science Center, Houston, Texas 77030, USA; 80 Medical Genomics and Metabolic Genetics Branch, National Human Genome Research Institute, NIH, Bethesda, Maryland 20892, USA; 81 Medical Faculty Carl Gustav Carus, Department of Medicine III, University of Dresden, 01307 Dresden, Germany; 82 University of Groningen, University Medical Center Groningen, The LifeLines Cohort Study, 9700 RB Groningen, The Netherlands; 83 Los Angeles BioMedical Resesarch Institute at Harbor-UCLA Medical Center, Torrance, California 90502, USA; 84 Department of Psychiatry, University of California, Los Angeles, California 90095, USA; 85 University of Sassari, 07100 Sassari, Italy; 86 EMGO Institute for Health and Care Research, VU University Medical Center, 1081 BT Amsterdam, The Netherlands; 87 Department of Nutrition and Dietetics, School of Health Science and Education, Harokopio University, 17671 Athens, Greece; 88 Institute of Epidemiology I, Helmholtz Zentrum München-German Research Center for Environmental Health, 85764 Neuherberg, Germany; 89 Institute of Epidemiology II, Helmholtz Zentrum München-German Research Center for Environmental Health, 85764 Neuherberg, Germany; 90 Department of Chronic Disease Prevention, National Institute for Health and Welfare, FI-00271 Helsinki, Finland; 91 Department of General Practice and Primary Health Care, University of Helsinki, FI-00014 Helsinki, Finland; 92 Institute of Clinical Chemistry and Laboratory Medicine, University Medicine Greifswald, 17475 Greifswald, Germany; 93 Hypertension and Related Disease Centre, AOU-University of Sassari, 7100 Sassari, Italy; 94 German Center for Diabetes Research (DZD), 85764 Neuherberg, Germany; 95 Research Unit of Molecular Epidemiology, Helmholtz Zentrum München-German Research Center for Environmental Health, 85764 Neuherberg, Germany; 96 Department of Medicine III, Pathobiochemistry, Technische Universitaet, 01307 Dresden, Germany; 97 Department of Medicine, Karolinska Institutet, Stockholm, Sweden; 98 Department of Medicine Solna, Atherosclerosis Research Unit, Karolinska Institutet, 17176 Stockholm 17176, Sweden; 99 Center for Molecular Medicine, Karolinska University Hospital, 17176 Stockholm, Sweden; 100 University of Groningen, University Medical Center Groningen, Interdisciplinary Center Psychopathology and Emotion Regulation, 9700 RB Groningen, The Netherlands; 101 Kuopio Research Institute of Exercise Medicine, 70100 Kuopio, Finland; 102 Institue of Biomedical & Clinical Science, University of Exeter, Barrack Road, Exeter EX2 5DW, UK; 103 Department of Public Health and General Practice, Norwegian University of Science and Technology, 7489 Trondheim, Norway; 104 School of Population Health, University of Western Australia, Nedlands, Western Australia 6009, Australia; 105 Research Centre for Prevention and Health, Glostrup Hospital, 2600 Glostrup, Denmark; 106 Department of Pulmonary Physiology and Sleep Medicine, Sir Charles Gairdner Hospital, Nedlands, Western Australia 6009, Australia; 107 Department of Clinical Medicine, Faculty of Health and Medical Sciences, University of Copenhagen, 2200 Copenhagen, Denmark; 108 Faculty of Medicine, University of Aalborg, 9220 Aalborg, Denmark; 109 Research Centre for Prevention and Health, Capital Region of Denmark, DK2600 Glostrup, Denmark; 110 Department of Clinical Physiology, Tampere University Hospital, FI-33521 Tampere, Finland; 111 Department of Clinical Physiology, University of Tampere School of Medicine, FI-33014 Tampere, Finland; 112 Echinos Medical Centre, 67300 Echinos, Greece; 113 Clinical Gerontology Unit, Box 251, Addenbrooke's Hospital, Hills Road, Cambridge CB2 2QQ, UK; 114 Faculty of Medicine, Institute of Health Sciences, University of Oulu, Oulu F1-90014, Finland; 115 Unit of General Practice, Oulu University Hospital, Oulu FI-90029, Finland; 116 National Institute for Health and Welfare, FI-00271 Helsinki, Finland; 117 Department of Medicine and Abdominal Center: Endocrinology, University of Helsinki and Helsinki University Central Hospital,, 00029 Helsinki, Finland; 118 Minerva Foundation Institute for Medical Research, 00290 Helsinki, Finland; 119 Department of Public Health, Faculty of Medicine, University of Split, 21000 Split, Croatia; 120 Kuopio University Hospital, 70029 Kuopio, Finland; 121 Department of Clinical Physiology and Nuclear Medicine, University of Eastern Finland, FI-70211 Kuopio, Finland; 122 Department of Epidemiology and Public Health, UCL, London WC1E 6BT, UK; 123 Institute of Human Genetics, Helmholtz Zentrum München-German Research Center for Environmental Health, 85764 Neuherberg, Germany; 124 The Big Data Institute, University of Oxford, Oxford OX3 7LJ, UK; 125 Department of Clinical Experimental Research, Rigshospitalet, 2600 Glostrup, Denmark; 126 Strangeways Research Laboratory Wort's Causeway, Cambridge CB1 8RN, UK; 127 Lund University Diabetes Centre and Department of Clinical Science, Diabetes & Endocrinology Unit, Lund University, 221 00 Malmö, Sweden; 128 Chair of Nephrology, Università Vita Salute San Raffaele and Genomics of Renal Diseases and Hypertension Unit, IRCCS San Raffaele Scientific Institute, Milan 20139, Italy; 129 School of Social and Community Medicine, University of Bristol, Bristol BS82BN, UK; 130 Divison of Public Health Sciences, Program in Biostatistics and Biomathematics, Fred Hutchinson Cancer Research Center, Seattle, Washington 98109, USA; 131 Department of Genetics, University of North Carolina, Chapel Hill, North Carolina 27599, USA; 132 Division of Endocrinology, Diabetes & Nutrition, Department of Medicine, Program for Personalized and Genomic Medicine, University of Maryland School of Medicine, Baltimore, Maryland 21201, USA; 133 Department of Biostatistics, University of Liverpool, Liverpool L69 3GA, UK; 134 Center for Evidence Based Healthcare, University of Dresden, Medical Faculty Carl Gustav Carus, Dresden, 01307, Germany; 135 Department of Respiratory Medicine, Sir Charles Gairdner Hospital, Nedlands, West Australia 6009, Australia; 136 Department of Paediatrics, University of Cambridge, Cambridge CB2 0QQ, UK; 137 MRC Lifecourse Epidemiology Unit, University of Southampton, Southampton General Hospital, Southampton SO16 6YD, UK; 138 Massachusetts General Hospital, Center for Human Genetic Research, Psychiatric and Neurodevelopmental Genetics Unit, Boston, Massachusetts 02114, USA; 139 Division of Epidemiology and Community Health, School of Public Health, University of Minnesota, Minneapolis, Minnesota 55455-0381, USA; 140 MRC Integrative Epidemiology Unit, School of Social and Community Medicine, University of Bristol, Bristol BS8 1TH, UK; 141 Department of Psychiatry and EMGO Institute for Health and Care Research, VU University Medical Center, AJ Ernstraat 1887, 1081 HL Amsterdam, The Netherlands; 142 Center for Biomedicine, European Academy Bozen/Bolzano (EURAC), 39100 Bolzano, Italy; 143 Affiliated Institute of the University of Lübeck, 23562 Lübeck, Germany; 144 Istituto di Ricerca Genetica e Biomedica, CNR, 9042 Monserrato, Italy; 145 Department of Neurology, University of Lübeck, 23562 Lübeck, Germany; 146 Department of Neurology, General Central Hospital, 39100 Bolzano, Italy; 147 Department of Clinical Physiology and Nuclear Medicine, Turku University Hospital, FI-20521 Turku, Finland; 148 Research Centre of Applied and Preventive Cardiovascular Medicine, University of Turku, FI-20520 Turku, Finland; 149 Human Genomics Laboratory, Pennington Biomedical Research Center, Baton Rouge, Louisiana 70808, USA; 150 Department of Psychiatry, Washington University School of Medicine, St. Louis, Missouri 63110, USA; 151 Wellcome Trust Sanger Institute, Human Genetics, Hinxton CB10 1HH, UK; 152 Harvard Medical School, Boston, Massachusetts, 02115, USA; 153 Biocenter Oulu, University of Oulu, Oulu FI-90014, Finland; 154 Center For Life-Course Health Research, University of Oulu, FI-90014 Oulu, Finland; 155 Institute of Medical Informatics, Biometry and Epidemiology, Chair of Genetic Epidemiology, Ludwig-Maximilians-Universität, 81377 Munich, Germany; 156 Anogia Medical Centre, 74051 Anogia, Greece; 157 Department of Endocrinology, University of Groningen, University Medical Center Groningen, 9700 RB Groningen, The Netherlands; 158 Institute of Ophthalmology, University College London, London EC1V 9EL, UK; 159 Department of Medicine, University of Turku, FI-20521 Turku, Finland; 160 Division of Medicine, Turku University Hospital, Turku, Finland; 161 School of Nutrition, Laval University, Québec, Québec, Canada G1V 0A6; 162 Department of Internal Medicine, University Hospital Lausanne (CHUV) and University of Lausanne, 1011 Lausanne, Switzerland; 163 Department of Community Medicine, Faculty of Health Sciences, University of Tromsø, 9037 Tromsø, Norway; 164 Imperial College Healthcare NHS Trust, London W12 0HS, UK; 165 Faculty of Health Sciences, University of Southern Denmark, 5000 Odense, Denmark; 166 Unit of Primary Care, Oulu University Hospital, 90029 OYS Oulu, Finland; 167 Department of Epidemiology and Biostatistics, MRC–PHE Centre for Environment & Health, School of Public Health, Imperial College London W12 0NN, UK; 168 Faculty of Medicine, Center for Life Course Epidemiology, University of Oulu, P.O.Box 5000, FI-90014 Oulu, Finland; 169 Children's Hospital, Helsinki University Hospital and University of Helsinki, FI-00029 Helsinki, Finland; 170 Department of Obstetrics and Gynecology, MRC Oulu, Oulu University Hospital and University of Oulu, FI-90029 Oulu, Finland; 171 National Heart and Lung Institute, Imperial College London, London W12 0NN, UK; 172 Department of Physiology, Institute of Biomedicine, University of Eastern Finland, Kuopio Campus, 70210 Kuopio, Finland; 173 Department of Clinical Chemistry, University of Tampere School of Medicine, FI-33014 Tampere, Finland; 174 Department of Clinical Chemistry, Fimlab Laboratories and School of Medicine, University of Tampere, FI-33520 Tampere, Finland; 175 Department of Clinical Medicine, Faculty of Health Sciences, University of Tromsø, 9037 Tromsø, Norway; 176 School of Public Health, University of Adelaide, Adelaide, South Australia 5005, Australia; 177 Robinson Research Institute, University of Adelaide, Adelaide, South Australia 5005, Australia; 178 DZHK (German Centre for Cardiovascular Research), partnersite Munich Heart Alliance, 80802 Munich, Germany; 179 Department of Medicine, University of Washington, Seattle, Washington 98101, USA; 180 Departments of Epidemiology and Health Services, University of Washington, Seattle, Washington 98101, USA; 181 Group Health Research Institute, Group Health Cooperative, Seatte, Washington 98101, USA; 182 South Ostrobothnia Central Hospital, Seinäjoki Fi-60220, Finland; 183 Department of Clinical Physiology and Nuclear Medicine, Kuopio University Hospital, 70211 Kuopio, Finland; 184 Paul Langerhans Institute Dresden, German Center for Diabetes Research (DZD), Dresden 01307, Germany; 185 Geriatric Research and Education Clinical Center, Vetrans Administration Medical Center, Baltimore, Maryland 21042, USA; 186 MRC Integrative Epidemiology Unit, School of Social and Community Medicine, University of Bristol, Bristol BS82BN, UK; 187 Institute of Preventive Medicine, Bispebjerg and Frederiksberg Hospital, The Capital Region, 2000 Frederiksberg, Denmark; 188 Faculty of Medicine, University of Iceland, 101 Reykjavik, Iceland; 189 Diabetes Prevention Unit, National Institute for Health and Welfare, FI-00271 Helsinki, Finland; 190 Centre for Vascular Prevention, Danube-University Krems, 3500 Krems, Austria; 191 Diabetes Research Group, King Abdulaziz University, Jeddah 21589, Saudi Arabia; 192 Department of Public Health and Clinical Nutrition, University of Eastern Finland, 70211 Kuopio, Finland; 193 Research Unit, Kuopio University Hospital, 70029 Kuopio, Finland; 194 DZHK (German Centre for Cardiovascular Research), Partner Site Greifswald, 17475 Greifswald, Germany; 195 Princess Al-Jawhara Al-Brahim Centre of Excellence in Research of Hereditary Disorders (PACER-HD), King Abdulaziz University, Jeddah 21589, Saudi Arabia; 196 Center for Medical Systems Biology, 2300 Leiden, The Netherlands; 197 Finnish Institute for Molecular Medicine (FIMM), Helsinki University, 00014 Helsinki, Finland; 198 Institute of Genetic Epidemiology, Helmholtz Zentrum München, Neuherberg 85764, Germany; 199 Department of Epidemiology, Harvard School of Public Health, Boston, Massachusetts 02115, USA; 200 Department of Epidemiology and Popualtion Health, Albert Einstein College of Medicine, Bronx, New York 10461, USA; 201 Oxford NIHR Biomedical Research Centre, Oxford OX3 7LJ, UK; 202 Department of Epidemiology, Carolina Center for Genome Sciences, University of North Carolina at Chapel Hill, Chapel Hill, North Carolina 27599-7400, USA; 203 National Institute on Aging, National Institutes of Health, Bethesda, Maryland 20892, USA; 204 Population Health Research Institute, St George's, University of London, London SW17 0RE, UK; 205 Genetics of Complex Traits, University of Exeter Medical School, University of Exeter, Exeter EX1 2LU, UK; 206 Metabolism Initiative, Broad Institute, Cambridge, Massachusetts 02142, USA; 207 Department of Medicine I, University Hospital Grosshadern, Ludwig-Maximilians-Universität, 81377 Munich, Germany; 208 The Genetics of Obesity and Related Metabolic Traits Program, The Icahn School of Medicine at Mount Sinai, New York, New York 10029, USA; 209 The Mindich Child Health and Development Institute, The Icahn School of Medicine at Mount Sinai, New York, New York 10029, USA

## Abstract

Large consortia have revealed hundreds of genetic loci associated with anthropometric traits, one trait at a time. We examined whether genetic variants affect body shape as a composite phenotype that is represented by a combination of anthropometric traits. We developed an approach that calculates averaged PCs (AvPCs) representing body shape derived from six anthropometric traits (body mass index, height, weight, waist and hip circumference, waist-to-hip ratio). The first four AvPCs explain >99% of the variability, are heritable, and associate with cardiometabolic outcomes. We performed genome-wide association analyses for each body shape composite phenotype across 65 studies and meta-analysed summary statistics. We identify six novel loci: *LEMD2* and *CD47* for AvPC1, *RPS6KA5*/*C14orf159* and *GANAB* for AvPC3, and *ARL15* and *ANP32* for AvPC4. Our findings highlight the value of using multiple traits to define complex phenotypes for discovery, which are not captured by single-trait analyses, and may shed light onto new pathways.

## Abstract (web summary)

Past genome-wide associate studies have identified hundreds of genetic loci that influence body size and shape when examined one trait at a time. Here, Jeff and colleagues develop an aggregate score of various body traits, and use meta-analysis to find new loci linked to body shape.

Large-scale meta-analyses of genome-wide association studies (GWAS) have identified numerous loci for anthropometric traits, including more than 600 loci for height^1^,^2^,^3^ and over 160 loci for obesity-related outcomes, predominantly for commonly available traits such as body mass index (BMI)^2^ and waist-to-hip ratio (WHR)^4^,^5^, but also for body fat percentage^6^, childhood obesity^7^ and extreme and early onset obesity^7^,^8^,^9^. While GWAS-meta-analyses have successfully revealed new loci, so far, all these studies have focused on one single anthropometric trait at a time and may not adequately capture differences in body shape between individuals who are similar in one trait but different in others. For example, two individuals may have the same BMI, but their WHR and/or height can differ substantially, so that each has a different body shape, which may translate into differences in disease risk^10^,^11^. Several loci identified from previous single-trait GWAS on BMI, BMI-adjusted WHR (WHRadjBMI) and height are associated with more than one anthropometric trait^1^,^2^,^4^,^12^. For example, the loci near *MC4R* and near *POMC/ADCY3* are each associated with BMI and height. However, the BMI-increasing allele of the near-*MC4R* locus is associated with increased height, whereas the BMI-increasing allele of the near-*POMC/ADCY3* locus is associated with reduced height^1^,^2^. Thus, these loci are likely each associated with a more comprehensive body shape phenotype that is not captured by current GWAS that only consider anthropometric traits individually.

In recent years, several approaches have been developed to examine whether single-nucleotide polymorphisms (SNPs) influence multiple correlated traits associated with disease^13^,^14^. However, most approaches test phenotypes separately and are thus subject to multiple testing penalties that ultimately reduce the statistical power to detect genotype–phenotype relationships among correlated traits. One way forward is to apply a dimension reduction method to the traits of interest, such as principal component analysis (PCA) that combines multiple correlated traits into a set of uncorrelated outcomes principal components(principal components (PCs))^15^,^16^. This method is very appealing to capture a composite phenotype, such as body shape. To date, no large-scale GWAS meta-analyses have been reported that aim to identify genetic loci associated with body shape based on simultaneous analysis of multiple anthropometric traits using PCA methods.

Therefore, the purpose of our study was twofold. First, we aimed to capture body shape in its multi-dimensional structure using PCs from several commonly available anthropometric traits. To allow the meta-analysis of summary statistics across a large number of cohorts, we developed an approach that calculates averaged PCs (AvPCs) that robustly represent body shape across a wide range of studies. Second, using this approach, we aimed to identify genetic loci associated with body shape based on the AvPCs in 65 studies of the GIANT Consortium, including >170,000 individuals.

## Results

### Defining composite phenotypes of body shape

**Figure 1:**
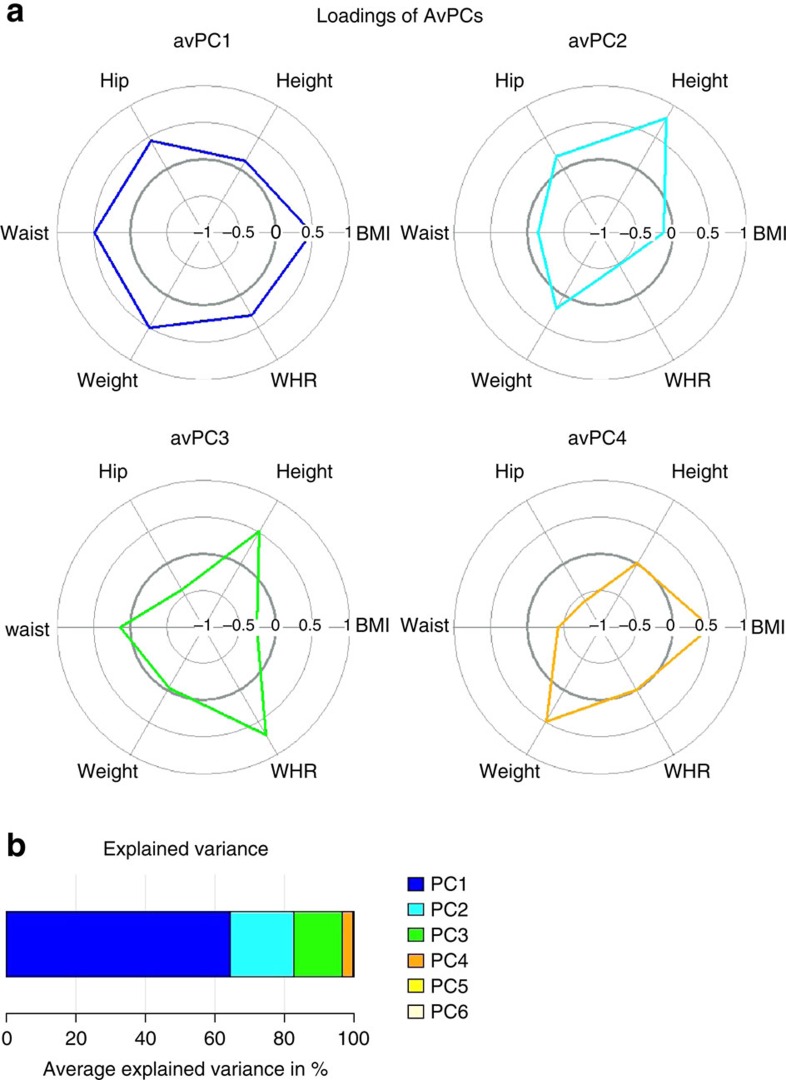
Loadings and explained variance of AvPCs for body shape. (**a**) Loadings of AvPCs, and (**b**) explained variance of AvPCs for body shape.

As basis for our analysis of body shape we used six anthropometric traits: BMI, WHR, height, weight, hip and waist circumference. First, we performed separate PCA in a subset of 20 large population-based studies (up to 82,355 individuals, Supplementary Table 1) and compared the loadings of the anthropometric traits in each PC between studies. Visual inspection of PCA loadings showed high concordance across studies (Supplementary Fig. 1) and between men and women. Between-study variation in variance explained by the PCs was small (Supplementary Fig. 1, Supplementary Table 2). On average, the first four PCs explained more than 99% of the variance (Fig. 1, Supplementary Table 2), and were therefore pursued as body shape outcomes for our gene-discovery effort. Given the across-study stability of PCs, we derived average loadings that were calculated as weighted means of loadings from all 20 population-based studies that were analysed in this step. We used these average loadings to calculate average principal components (AvPCs) as targets in each of the GWAS included in the first and second stage. In other words, the phenotypes used for genome-wide association were constructed in a consistent way across studies, such that the summary statistics could be meta-analysed.

Each AvPC represents a specific composition of the six anthropometric traits and thus captures a specific aspect of body shape (Fig. 1). The first AvPC, which explains on average 64.4% of the variation in all traits, shows high loadings for all traits, except for height. The loadings are in the same direction; meaning that the AvPC captures inter-individual variation in either increased or decreased BMI, weight, WHR, hip and waist circumference. Therefore, variation in this PC seems to predominantly capture overall adiposity. The second AvPC, which explains 18.5% of the variation, is characterized by particularly high but opposite loadings on height and WHR. In other words, AvPC2 captures variation in a composite phenotype that represents tall individuals with a small WHR or, vice versa, short individuals with a large WHR. The third AvPC, explaining 13.8% of the variation, also shows predominantly high loadings on height and WHR but in the same direction, with an opposite loading of nearly the same size on hip circumference. Given these loadings, AvPC3 discriminates mainly between tall individuals with a high WHR resulting from a smaller hip circumference on one extreme and short individuals with low WHR, and a larger hip circumference on the other extreme. The fourth AvPC explains on average 3% and is harder to interpret. It displays high loadings on BMI and body weight, and opposite loadings of a similar size on hip and waist circumference. These could be interpreted as a phenotype ranging between high BMI and weight, with relatively small hip and waist circumference on the one hand and low BMI and weight but large waist and hip circumference on the other hand.

Consistent with the individual anthropometric traits, the four AvPCs that describe body shape are also heritable. Using data from four isolate populations (*n*=4,000), we estimated that AvPC2 has the highest heritability (75–80%), consistent with the fact that height is the main contributing trait to this AvPC with a strong genetic component^1^. The heritability of AvPC1 (35–50%), AvPC3 (50–75%) and AvPC4 (25–50%) were moderately high and similar to the heritability for individual anthropometric traits^17^ (Supplementary Fig. 2). From a clinical perspective, each of the four AvPCs exhibit known correlations with cardio-metabolic traits (Supplementary Fig. 3), including diastolic blood pressure, systolic blood pressure, total cholesterol, low-density lipoprotein cholesterol, high-density lipoprotein cholesterol and total triglycerides levels.

### Genomic discovery of body shape composite phenotypes

**Figure 2:**
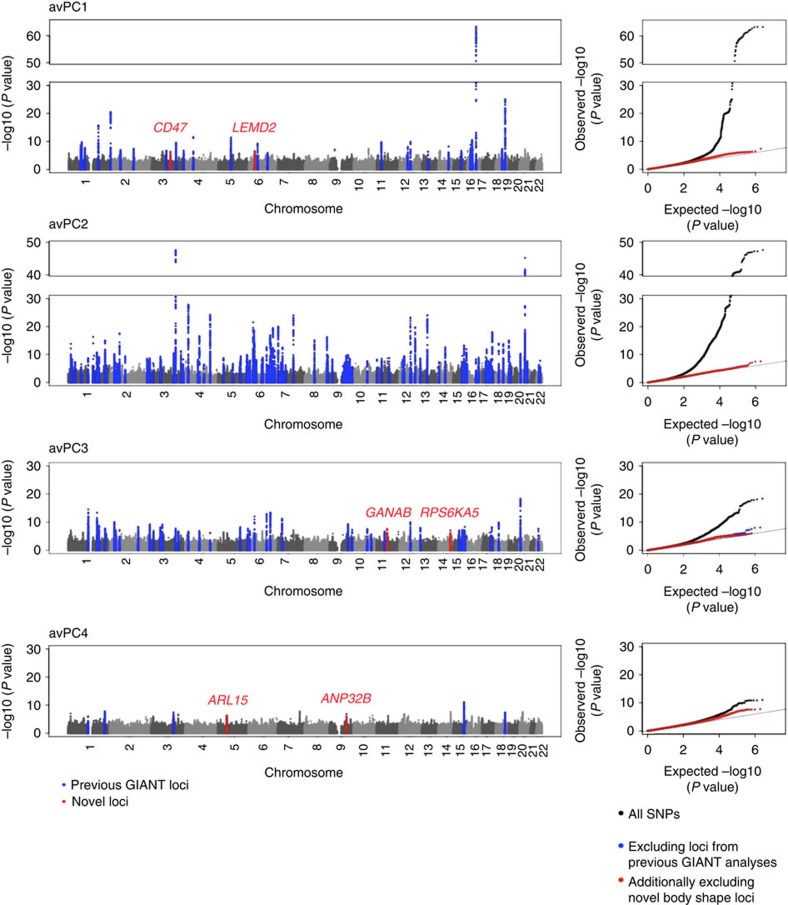
Manhattan and QQ-plots of association results on AvPCs of body shape. *P* values of the first stage meta-analysis are given in the Manhattan and QQ-plots. All genome-wide significant loci are highlighted.

**Figure 3:**
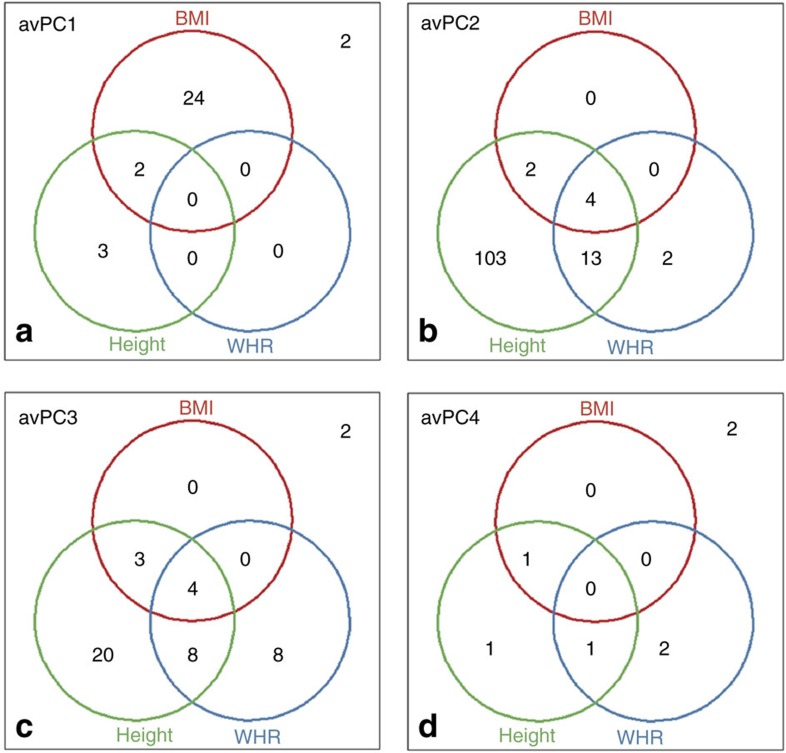
Number of loci associated with AvPCs and known from previous GIANT analyses on BMI, WHR or height. (**a–d**) corresponds to each AvgPC respectively. The Venn diagrams specify for each AvPC how many significantly associated loci (promising *P* value in the first stage meta analysis (<5 × 10^−6^) and genome wide significant in first and second stage combined analysis (<5 × 10^−8^)) are known from previous GIANT analysis on BMI, height or WHR. In the upper right corner of each plot the number of loci is given that are not known from previous GIANT analyses.

We performed a two-staged meta-analysis to identify genetic loci that are associated with the four AvPCs (Supplementary Table 3, Supplementary Table 4). In the first stage, a meta-analysis of 43 studies with imputed genome-wide SNP data including more than 133,000 individuals identified SNPs in 385 loci across the four AvPCs (56 loci for AvPC1, 205 for AvPC2, 89 for AvPC3 and 35 for AvPC4) that showed promising association (*P* value<5 × 10^−6^) for at least one of the four AvPCs (Fig. 2, Supplementary Fig. 4). Lead SNPs (and proxies; see ‘Methods' section) of each locus were taken forward for validation in a second stage, including data from more than 39,900 individuals from 22 studies of which 12 studies had genotypes from the Illumina CardioMetabochip and 10 studies had imputed genome-wide SNP data. In the combined analyses, consisting of the first and second stage studies, the association of 207 of the 385 loci reached genome-wide significance (*P* value <5 × 10^−8^) (31 for AvPC1, 124 for AvPC2, 45 for AvPC3 and 7 for AvPC4; Fig. 2, Fig. 3, Supplementary Fig. 4, Supplementary Table 6), of which 16 loci were identified for two AvPCs and one showed significant association with three AvPCs (Supplementary Fig. 7, Supplementary Table 5) resulting in a total of 189 loci with association to at least one AvPC. To determine whether the loci we identified were independent of the loci previously found for BMI, WHRadjBMI and height, we performed conditional analyses on SNPs reported in previous GIANT-GWAS publications on BMI, WHRadjBMI and height^1^,^2^,^4^,^5^,^18^,^19^. A locus was considered independent of reported findings if the *P* value in the analyses conditioned on all previously identified loci remained suggestive (*P* value <5 × 10^−6^). In total, 183 loci had already been established for BMI, WHRadjBMI or height (Fig. 3, Supplementary Fig. 7), whereas six loci had not previously been identified for association with conventional anthropometric traits; two for AvPC1, two for AvPC3 and two for AvPC4 (Table 1, local association plots given in Supplementary Fig. 5). For these six novel loci, the results of the lead SNPs were checked in previously performed GWAS meta-analyses on anthropometric and cardio-metabolic traits (Supplementary Table 7).

### Results for AvPC1

For AvPC1, we identified 31 genome-wide significant loci, of which two were novel (upstream of *LEMD2* and *CD47*). Of the 29 previously established loci, 24 have been associated with BMI only^18^, 3 with height only^1^,^3^, while two loci have been reported for associations with both BMI and height^3^,^18^ (Fig. 3a). While both novel loci showed some evidence of association with BMI in the latest GIANT–GWAS (*n*>339,000; *P*<7.2 × 10^−3^; Table 1), they did not reach genome-wide significance. The lead SNP (rs943466) 7 kb upstream of *LEMD2* has been reported to be associated with expression of *LEMD2* in liver (*P*=1.66 × 10^−9^) 20,21. Another variant in *LEMD2 (*rs2296743 at 8 kb from our lead SNP rs943466; *r*^2^=0.2, D′=1.0) was previously reported for its promising association (*P* value=8 × 10^−6^) with energy intake at dinner in a small GWAS of 815 Hispanic children^22^. The lead SNP (rs7640424) for the second novel locus was located in an enhancer region 10 kb upstream of *CD47* (refs 23, 24), which encodes a membrane protein that might be involved in signal transduction and membrane transport^25^. No genome-wide significant associations have been reported for the lead SNP or other SNPs in the *CD47* gene before^23^,^24^,^25^. However, a recent study revealed a link to diet-induced obesity in mice and suggests *CD47* as a potential drug-target to combat obesity and metabolic complications^26^,^27^.

### Results for AvPC2

For AvPC2, we identified no novel loci. Almost all (*n*=122) of the 124 loci associated with AvPC2 had previously been identified for height^1^ (Fig. 3b), which is consistent with AvPC2's high loadings on height and opposite loadings on WHR. Of these 122 loci, 103 were reported for association to height only, whereas of the 19 remaining loci, 4 were previously associated with height, BMI and WHRadjBMI, 2 loci were reported for height and BMI and 13 loci overlapped with height and WHR. The two AvPC2 loci that did not associate with height were previously identified for WHRadjBMI^19^.

### Results for AvPC3

We identified 45 loci that reached genome-wide significance for AvPC3, of which 2 were novel. Consistent with the loadings of AvPC3, 43 of the associated loci had been reported before for height^1^ or WHR^4^,^19^ (Fig. 3c). The lead SNP of the first novel locus rs7492628, upstream of the genes *RPS6KA5* (>20 kb) and *C14orf159* (>30 kb), failed to reach genome-wide significance in previous WHRadjBMI GWAS (*P* value=9.3 × 10^−8^) and was nominally associated with extreme obesity risk (*P* value=7.26 × 10^−5^) 28. The lead SNP of the other novel locus, *GANAB,* rs7949030, showed some evidence of association with WHRadjBMI in the latest GIANT GWAS (*P* value=3.3 × 10^−6^) and was reported to be an eQTL for several other genes^21^: In monocytes, regulation of *MIR3654*, *EEF1G*, *EML3*, *BSCL2*, *HNRNPUL2-BSCL2*, *LRRN4CL* was found^29^,^30^,^31^. *BSCL2* is of interest, as it is a known candidate gene for the most severe lipodystrophy phenotype^32^. In blood rs7949030 was found to be an eQTL of *HNRNPUL2-BSCL2*, *AHNAK*, *LRRN4CL* and *INTS5* (refs 33, 34), while in skin and adipocytes it was found as an eQTL for *EML3* (refs 30, 31, 35).

### Results for AvPC4

Seven loci were identified for AvPC4, of which five had been previously reported; one for BMI and height, one for WHR and height, one for height only and two for WHR only^1^,^3^,^4^,^36^ (Fig. 3). The lead SNPs of the two novel loci identified with AvPC4 were both intronic, in *ARL15* and *ANP32*. The allele associated with increased AvPC4 of the lead SNP (rs4865796) in *ARL15* was moderately associated with higher BMI (*P* value=1.6 × 10^−4^), increased adiponectin levels (*P* value=4.2 × 10^−6^ ADIPOGEN^37^) and decreased risk of diabetes (*P* value=1.8 × 10^−5^, DIAGRAM^38^). This SNP was associated with fasting insulin (rs4865796, *P*=2.1 × 10^−8^ and 2.2 × 10^−12^ after adjustment for BMI^39^). Other nearby SNPs in high linkage disequilbrium (LD), have previously been reported for associations with BMI-adjusted adiponectin levels (rs6450176/rs4311394, *r*^2^=0.087, D′=0.87 (refs 37, 40)), high density lipoprotein C (HDL-C) levels (rs6450176 (refs 41, 42)) and risk of type 2 diabetes (rs702634, *r*^2^=1.0, D′=1.0 (ref. 38)). A duplication in *ARL15*, tagged by rs16992296) was previously found to be associated with increased risk of childhood obesity in European and African Americans^43^. However, this duplication is independent of the association we found for rs4865796-ARL15 and AvPC4, which is in low LD (*r*^2^EUR=0.065) with the duplication (represented by rs16992296), located 168 kb upstream. The lead SNP (rs7855432) of the second locus, *ANP32B,* was moderately associated with height (*P* value=5.5 × 10^−6^) 1. A SNP in high LD (rs4743150 *r*^2^=0.95, D′= 1.0) was reported to be promisingly associated with coronary heart disease risk (*P* value=5 × 10^−6^)^44^.

## Discussion

We developed a PCA-based approach to capture variation across multiple traits simultaneously in a uniform way across multiple studies. Resulting AvPCs are a robust cross-phenotype representation allowing their use in large-scale meta-analyses. We assessed this approach to capture body shape based on six individual anthropometric traits and identified six novel loci that were not identified before in much larger GWAS-meta-analyses for BMI, WHRadjBMI and height^1^,^2^,^4^. Our findings suggest that the body shape composite phenotype, assessed by AvPCs, represents information that is not fully captured by individual (anthropometric) traits. Application of this method to other related traits, for example, in immune disease, different types of cancer, cardiometabolic traits, or other correlated traits might comparably reveal new loci, and potentially new pathways, that have not been identified in single-trait GWAS.

The AvPCs are combinations of different anthropometric traits and therefore capture more complex body shape phenotypes than the single traits. AvPC1, representing overall adiposity, and AvPC2, representing height with respect to WHR, are the most important contributors to body shape, explaining on average more than 80% of the variation. More specific body shape types were captured by AvPC3 and AvPC4 and were defined by impact of height and WHR (AvPC3) or BMI, waist and hip (AvPC4). Our initial analyses demonstrated that the loadings are stable across studies, study designs and between men and women. Moreover, we have shown that the AvPCs are heritable traits and correlated with cardiometabolic traits and risk factors.

To further demonstrate the strength of this approach, we compared total variance explained of single traits and AvPCs by SNPs previously identified in single-trait GWAS (for BMI, WHRadjBMI, height^1^,^2^,^4^). For example, the 97 loci that have been reported for association in the latest BMI single-trait GWAS (N∼340,000) explain 8.7% of the variation in AvPC1, whereas they explained only 2.68% of the variation in BMI^2^. These data indicate that our PC-defined phenotype for overall body size (AvPC1) captures a more composite phenotype compared with BMI as a single-trait. Explaining more of the variance with the same genetic variants as previous single-trait studies in our composite phenotype shows promise to update and inform existing methods.

So far, typical GWAS have tested for association of genetic variants with anthropometric traits, one trait at a time. We define ‘body shape' as a composite of multiple traits defined by PCs. We first performed PC-analyses in representative population-based studies and averaged PC loadings across these studies (AvPCs). We subsequently use these AvPCs to calculate PCs in all participating studies. This approach ensures that PCs are calculated in a uniform manner across all studies, thus facilitating subsequent meta-analyses. This approach could be applied to capture genetic variation across related traits that is currently not captured by single-traits GWAS (for example, in the context of autoimmune disease, blood traits, lipid levels, different cancers and so on.).

Consistent with published anthropometric traits^10^,^11^,^17^, the derived AvPCs are heritable and correlated with clinically relevant outcomes. We identified additional loci, despite a much smaller sample size compared with the latest single-trait GWAS analyses for BMI, height and WHRadjBMI^1^,^2^,^4^. This suggests that the AvPC method captures phenotype information that is not captured by single-trait analyses and associated loci may highlight biological pathways that are not revealed with single-trait associated loci only.

Even though our approach has several advantages, it is not meant to replace single trait GWAS analyses. A number of loci that were identified in the latest single-trait GWAS were not identified in our body shape GWAS; that is, we identified 124 loci (or 14.2%) of the 837 loci recently reported in the GIANT single-trait meta-analyses (Supplementary Fig. 6). This may be due to the fact that these recent single-trait GWAS meta-analyses were at least twice as large as the current body shape GWAS. However, even when we compare the number of identified loci in earlier GWAS meta-analyses, which are of similar size as the current body shape GWAS, we do not identify all previously reported loci for single traits. Perhaps this is most obvious with height (largely representative of AvPC2), where we only identified 91 (13.1%) of 697 loci identified for height. This is in part due to the fact that a conservative definition for linkage disequilibrium was applied (*r*^2^>0.8), lack of power due to sample size for SNPs of modest effects, or perhaps the AvPCs introduces noise to purely single traits such as height. Consistent with this finding, we also observe that some single traits also explain more of the variance of body shape compared with AvPCs. Our comparison of the variance explained between previous single-traits meta-GWAS and our AvPCs support this evidence for overlapping associated variants. Since AvPC2 represents largely a single trait, height, with large height loadings we were unable to explain more of the variance. In fact we explained less of the variance, which is likely due to noise introduced using this composite AvPCs phenotype. This observation is also evident for variance in body shape explained by height compared with AvPC3 and AvPC4, but is in contrast to BMI, a complex trait comprised of multiple anthropometric measurements, which explains less variance in body shape compared with AvPC3 and AvPC4. It is important to emphasize our approach is most informative for complex traits such as BMI that are derived from a series of other traits. We believe that using PC space to define complex traits is useful for the detection of loci involved in multiple pathways that might go undetected in a single trait setting.

We have developed a new strategy that applies a PCA approach in a meta-analysis setting to combine composite phenotypes in a harmonized way across multiple studies. We successfully applied this approach to anthropometric traits to capture body shape. The derived combined anthropometric traits (AvPCs) were shown to be heritable and correlated to cardio-metabolic traits. Large-scale GWAS meta-analyses of the AvPCs identified six new loci that were not identified by previous single-trait GWAS that were twice as large in samples size. This PCA approach could maximize gene discovery for other correlated traits, such as cancers, immune disease, hematologic traits and so on. and may identify genes that point towards shared physiological pathways.

## Methods

### Study description

In the first stage analyses, 43 studies participated (133,376 individuals) that had HapMap 2 imputed genome-wide data available. A subset of 20 studies with unrelated individuals was used for calculation of average loadings. Second stage analyses were performed in 10 studies (7,734 individuals) with genome-wide data that became available after the first stage and 12 studies (32,170 individuals) with Cardio-MetaboChip (by Illumina) data (number of included studies and individuals given in Supplementary Table 3). Details on study phenotypes, genotyping and imputation of each study are given in the Supplementary Tables 8 and 9, respectively.

### Ethics statement

All study participants gave written informed consent and ethic committees approved all studies. The ethic statement of each study is given in the study specific acknowledgements.

### Calculation of average loadings

In 20 independent studies (Supplementary Table 1) with unrelated participants PCAs were performed on six anthropometric traits (BMI, height, hip, waist, weight and WHR). Each study performed a PCA on the standardized residuals of the anthropometric traits adjusted for age and gender. The same analyses were done for men and women separately with residuals adjusted for age only. The result of the PCA in each study is a set of six PCs that are orthogonal linear combinations of the six anthropometric traits. In other words each PC is a weighted sum of the six transformed anthropometric traits and independent of the other PCs. The weights of each trait per PC are called loadings. Each study also calculated the explained variance per PC. The loadings and explained variances were comparable for all studies (Supplementary Fig. 1 (1)).

With the intention to create phenotypes that are identically constructed in all studies, the results of single study PCAs were used to deduce the average loadings. This approach is reasonable as the loadings of the study specific PCAs were comparable. With the use of the single study correlation matrices a combined average correlation matrix was derived (weighted sum divided by number of individuals). This average correlation matrix is then used as basis for a PCA. The loadings that result from this PCA are called average loadings (Fig. 1a) and Supplementary Table 2). This was performed for men, women and all individuals combined, however ultimately we used combined loadings for primary results reported in the manuscript. Sex specific results are reported in the Supplementary Material. The average loadings and explained variance were comparable to the study specific loadings and explained variances (Supplementary Fig. 1).

### Heritability analyses

Heritability of the avPCs was calculated within four population isolates, CROATIA-Vis (*n*=909), CROATIA-Korcula (*n*=842), CROATIA-Split (*n*=499) and ORCADES (*n*=866) using the ‘polygenic' function of the GenABEL package for R 45.

### Average principal components as body shape phenotype

The average loadings were used in each study to calculate the AvPCs in a standardized way. Therefore, the average loadings were distributed together with an R-script (http://www.r-project.org/) that calculated the AvPCs as linear combination of residuals of the study phenotypes with the use of the average loadings. This was done for men and women separately and additionally for combined in studies with relatedness structure. As the first four PCs explain on average more than 99% of the variance (Fig. 1b) we decided to limit all analyses to these four PCs.

### Stage 1 analyses

GWAS on the first four AvPCs were calculated for men and women separately in studies of unrelated samples and combined for studies with related samples with an adjustment for study site when necessary. All studies of the first stage analyses used HapMap 2 imputed genome-wide data. GWAS results underwent extensive quality control and study-wise filtering (call rate >95%, *P* value (HWE)>10^−6^, imputation quality, minor allele count (MAC) >3). The meta analyses of GWAS results for the first four AvPCs we combined sex-stratified results for studies with unrelated individuals and unstratified GWAS results for studies with relatedness individuals. Meta analyses were performed with METAL 46 using fixed effects inverse variance-weighted method. Single study and the meta analysis *P* values were corrected by the genomic control inflation factor *λ* (meta analysis *λ* before correction: *λ*(PC1)=1.29, *λ*(PC2)= 1.407, *λ*(PC3)= 1.236, *λ*(PC4)= 1.136). Results were limited to SNPs that are in HapMap 2 and had results for more than 30,000 individuals. Heterogeneity analysis was performed with METAL. Each AvPC all SNPs with a promising *P* value (*P* value<5 × 10^−6^) were identified in combined analyses. To identify promising loci clustering (LD>0.01, distance <1,000 kb) with PLINK^47^ based on HapMap 2 genotypes was performed. All leading SNPs per clump for AvPCs were taken forward to second stage analyses and named promising SNPs in this manuscript.

Two SNPs that were promising for the first principal component had very low heterogeneity *P* values (rs10847678 (*P* value(het)=8.8 × 10^−152^), rs13296358 (*P* value(het)=5.4 × 10^−67^)). For both SNPs the effect was driven only by a single study and no other SNP in high LD had a promising *P* value. Therefore, these two SNPs were removed from further analyses.

### Stage 2 Analyses

As mentioned above for second stage analyses a mixture of studies with genome-wide SNP data and MetaboChip genotypes was available. Some of the leading SNPs of the first stage analyses were not genotyped on the MetaboChip. To increase the power for all promising SNPs of each AvPC proxies were defined that were all SNPs close to promising SNPs (distance <500 kb), in high LD (LD>0.9) and available in more than 70% of the individuals of the second stage. Results of the second stage analyses underwent the same quality control as first stage results.

### Combined analyses

The combined analyses of all first and second stage GWAS was performed with METAL^35^ with inverse variance based method. Results for men and women were combined as described for the first stage meta-analyses. All promising loci for which at least one proxy had a genome-wide significant *P* value in the combined analysis were named genome-wide significant loci and the best SNP of the combined analyses (largest absolute beta) was reported as topSNP of this locus.

### Novel loci - conditional analyses and look-ups in previous GIANT analyses

Two analyses were performed to distinguish between genome-wide significant body shape loci that are known from previous GWAS on BMI, height and WHR and novel body shape loci. First, conditional analyses were performed. We used the 226 reported topSNPs (32 BMI, 180 height, 14 WHR) of published GIANT analyses on BMI, height and WHR^1^,^2^,^4^ to perform conditional analyses of the first stage meta-analyses using GCTA^15^,^48^. The results of this analysis were then analysed conditioned on 843 topSNPs (97 BMI, 697 height, 49 WHR) of the published GIANT analyses^1^,^2^,^4^. To identify the overlap of the results for AvPCs with the single anthropometric traits, the same conditional analyses were performed for BMI, height and WHR separately. For calculation of the LD-structure genotype data from KORA F4 was used. Two topSNPs of the unpublished GIANT results had to be removed before analyses as they were in high correlation with two other topSNPs. If the body shape topSNPs were independent loci identified by previous GIANT analyses, the *P* value should stay promising (*P* value<5 × 10^−6^) in both conditional analyses. Second, we checked by look-ups if those genome-wide significant SNPs that are independent from the previously reported topSNPs were not genome-wide significant (*P* value>5 × 10^−8^) in GIANT analyses^1^,^2^,^4^.

Genome-wide significant SNPs are named *novel SNPs* if they fulfil the following conditions:

- *P* value of conditioned analyses on topSNPs reported by previous GIANT analyses (on BMI, height, WHR) remained promising (*P* value<5 × 10^−6^).
- *P* value in previous GIANT analyses (on BMI, height, WHR) was not genome-wide significant (*P* value>5 × 10^−8^).

### Pleiotropic effects

For identification of potential pleiotropic effects several look-ups in various large-scale consortia on different phenotypes were performed, including GIANT, DIAGRAM and MAGIC, all references are given in the results table of the look-ups (Supplementary Table 7). For comparison of effect directions the loadings of each AvPC have to be considered. For example AvPC2 includes height with a positive loading and BMI with a negative loading. That means an increasing effect on AvPC2 means an increasing effect on height but a decreasing effect on BMI.

### Further Analyses

PCA, further analyses and plots were generated with R (http://www.r-project.org/) if not stated otherwise. Apart from the GCTA analyses, which uses LD structure of KORA F4, all LD analyses were performed in PLINK based on HapMap 2 (CEU) genotypes. For comparison of findings between loci from different AvPCs two loci are assumed to be identical if the topSNPs are in high LD (LD>0.8).

### Data availability

Summary statistics of all analyses can be downloaded from: https://www.broadinstitute.org/collaboration/giant/

## Additional information

**How to cite this article**: Ried, J. S. *et al.* A principal component meta-analysis on multiple anthropometric traits identifies novel loci for body shape. *Nat. Commun.* **7**, 13357 doi: 10.1038/ncomms13357 (2016).

**Publisher's note:** Springer Nature remains neutral with regard to jurisdictional claims in published maps and institutional affiliations.

## Supplementary Material

Supplementary Information Supplementary Figures 1-7, Supplementary Tables 1-5

Supplementary Data 1 Association results for 189 GWAS-significant loci.

Supplementary Data 2 Transferability of novel loci in other anthropometric GWAS

Supplementary Data 3 Phenotype summary statistics of all studies in 1st and 2nd stage analyses.

Supplementary Data 4 Genotyping description for all studies in 1st and 2nd stage analyses.

## Figures and Tables

**Table 1:** Association results for novel loci with avPC of body shape.

					**1st stage up to 133,376 samples**	**2nd stage up to 39,904 samples**	**1st+2nd stage combined up to 173,278 samples**			**Conditioned analysis on all GIANT tophits**		***P*** **value of SNPs in GIANT analysis**†			***P*** **value of SNPs in GIANT analysis**‡		
**trait**	**SNP (lead SNP)**	**Next gene**	**Effect/other allele**	EAF*	***P*** **va**l**ue**	***P*** **value**	**beta (sebeta)**	***P*** **value**	***N***	**beta (sebeta)**	***P* value**	**BMI**	**Height**	**WHR**	**BMI**	**Height**	**WHR**
avPC1	rs7640424	CD47	C/T	69%	5.40E-07	0.0015	0.05 (0.008)	3.18E-09	171,544	0.05 (0.01)	5.80E-07	0.0072	0.74	0.25	2.28E-06	0.28	0.85
avPC1	rs943466 (rs2281819)	LEMD2	G/A	76%	6.39E-07	0.016	0.049 (0.009)	3.47E-08	172,174	0.049 (0.01)	7.28E-07	2.7E-04	0.045	0.54	9.34E-06	0.75	0.25
avPC3	rs7949030	GANAB	G/A	38%	2.74E-08	0.11	0.024 (0.004)	5.58E-09	139,195	0.025 (0.004)	6.36E-09	0.082	0.80	1.4E-04	0.54	0.041	3.3E-06
avPC3	rs7492628	RPS6KA5	G/C	30%	8.75E-08	0.13	0.024 (0.004)	1.90E-08	139,874	0.024 (0.004)	7.93E-08	0.064	0.62	4.9E-05	0.0050	0.58	9.3E-08
avPC4	rs4865796 (rs1664781)	ARL15	G/A	32%	5.59E-07	0.011	0.008 (0.001)	2.25E-08	172,517	0.008 (0.002)	7.25E-07	5.1E-05	0.034	0.40	1.6E-04	0.020	0.84
avPC4	rs7855432	ANP32B	G/T	80%	1.40E-07	0.17	0.01 (0.002)	4.06E-08	140,805	0.01 (0.002)	1.78E-07	0.33	0.046	0.49	0.32	5.5E-06	0.91

The association results for the first stage, second stage and first and second stage combined analysis is given for all six loci that were genome wide significantly associated (promising *P* value in the first stage meta analysis (<5 × 10^−6^) and genome wide significant in first and second stage combined analysis e (<5 × 10^−8^)) with one of the avPCs and novel. Moreover, the *P* values of the analysis conditioned on all tophits from the recent GIANT publications on BMI, height and WHR.

^*^EAF is mean of EAF of all studies in the first stage meta analysis.

^†^All tophits of the GIANT analysis published before 2014 (refs 3, 6).

^‡^All tophits of the GIANT analysis unpublished and/or published after 2014 (refs 1, 2, 4).
