# Supplementary material for: A principal component meta-analysis on multiple anthropometric traits identifies novel loci for body shape
Source: Nat Commun. 2016 Nov 23;7:13357. doi: 10.1038/ncomms13357 (PMC5114527; doi:10.1038/ncomms13357)
Supplement: Supplementary Information — Supplementary Figures 1-7, Supplementary Tables 1-5 [file ncomms13357-s1.pdf]

Supplementary Figures

Supplementary Figure 1: Results of the pilot analysis.

(1) Study specific loadings for the PCs in the pilot analysis.

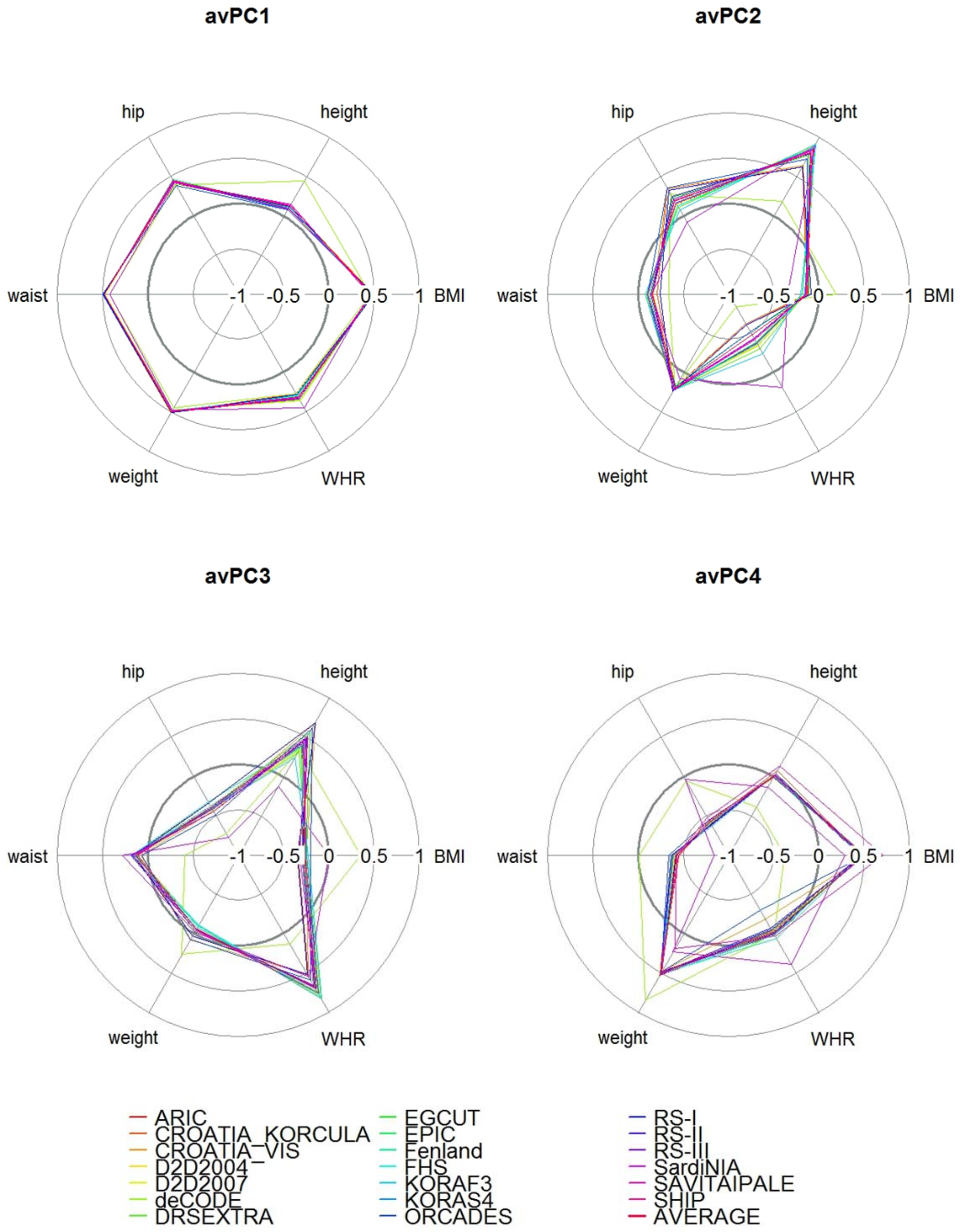

(2) Explained variance of PCs in each study of the pilot analysis.

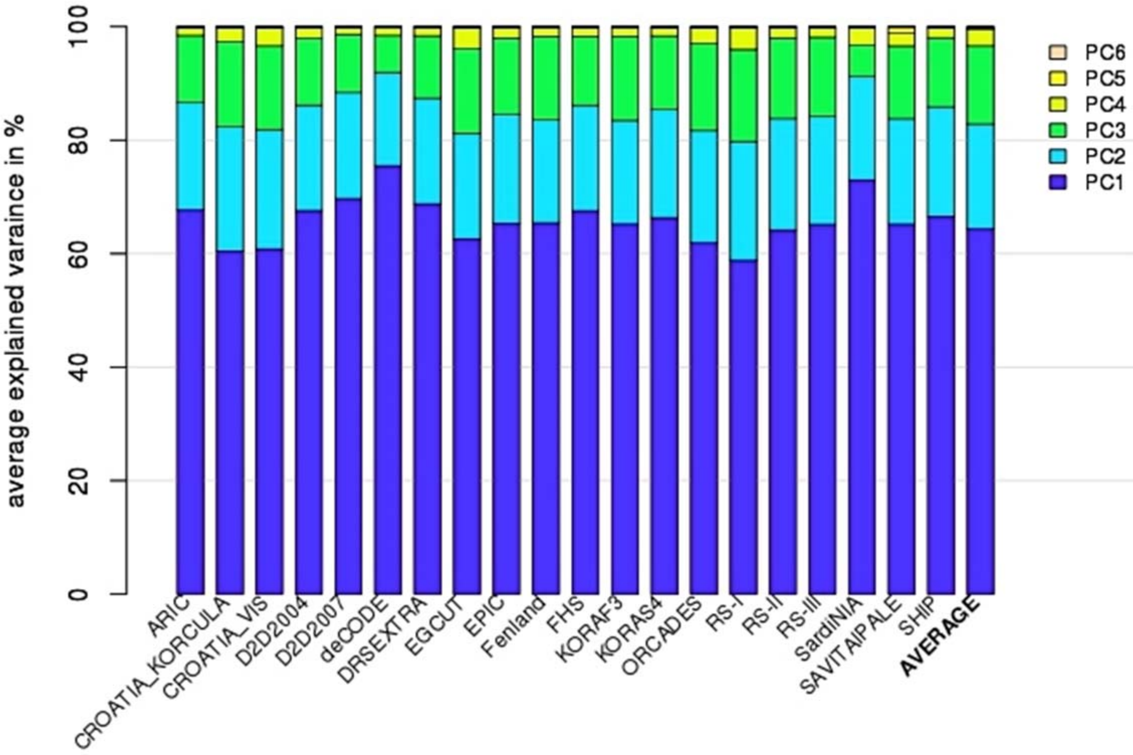

Supplementary Figure 2: Heritability of AvPCs.

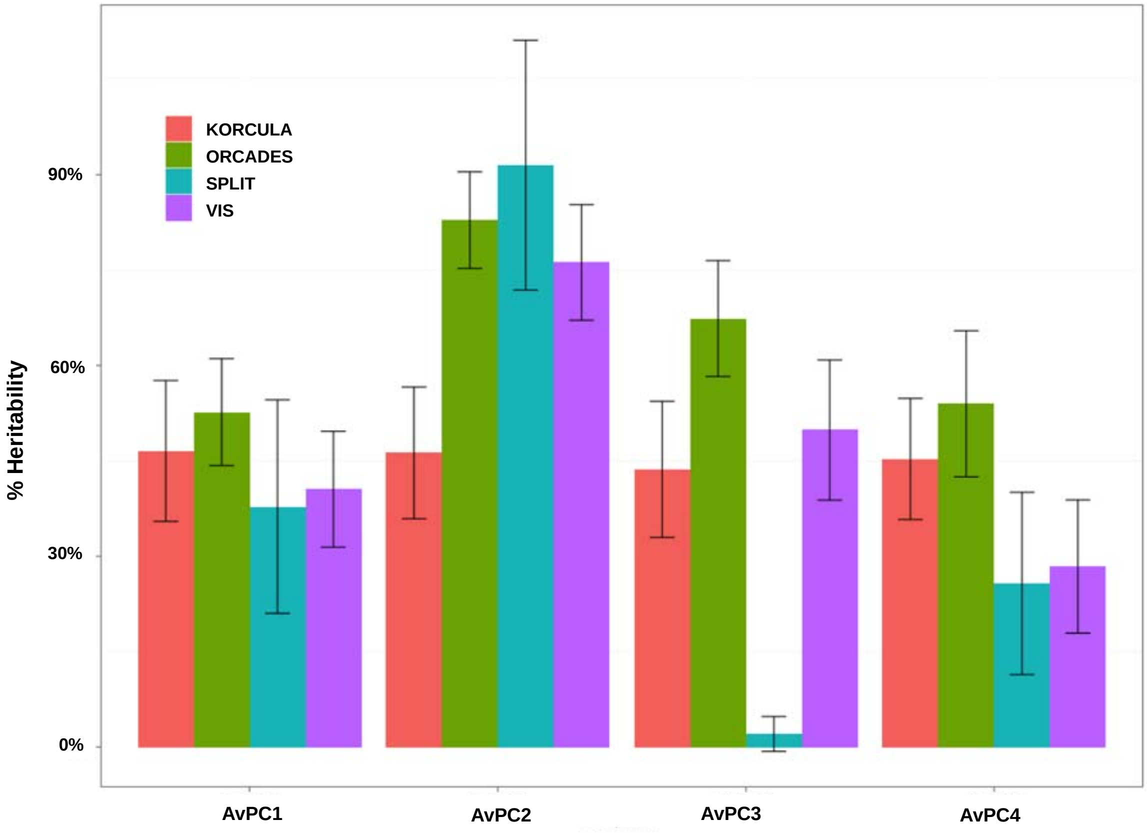

Supplementary Figure 3: Correlation of avPCs with clinical traits. Association results for n=1,402 persons from the from FENLAND study.

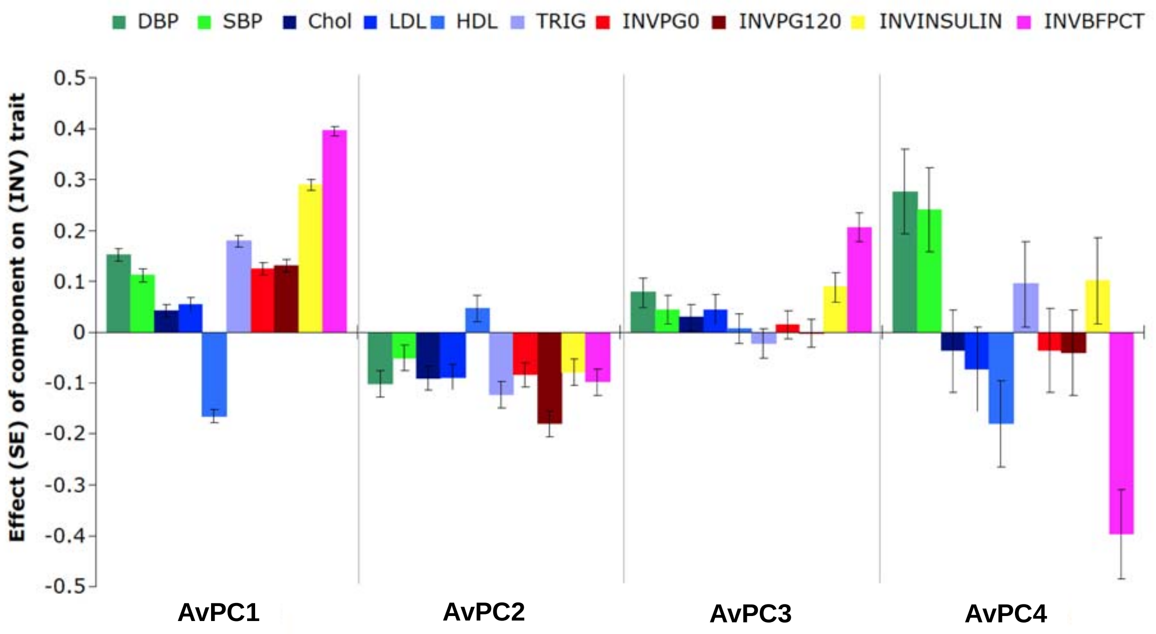

Supplementary Figure 4: Phenograms of all 189 genome-wide significant loci for avPCs. All genome wide significantly associated loci (promising p-value in the first stage meta analysis ($<5x10^{-6}$) and genome-wide significant in first and second stage combined analysis ($<5x10^{-8}$)) with one of the avPCs. Different colours are used for the four avPCs. Novel loci are highlighted by a diamond shape and the name of the nearest gene. (PhenoGram was generated with the online tool: http://visualization.ritchielab.psu.edu/phenograms/plot.)

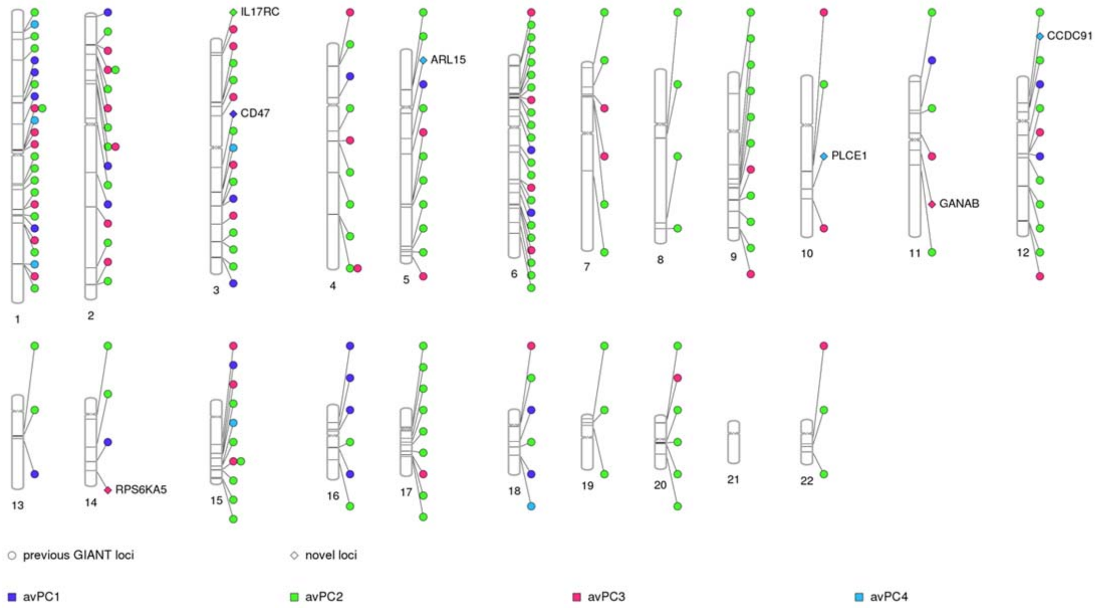

Supplementary Figure 5: Local association plots for novel loci. Local association plots of the six loci that were significantly associated (promising p-value in the first stage meta analysis (<5x10^{-6}) and genome-wide significant in first and second stage combined analysis (<5x10^{-8})) with an avPC of body shape and independent of findings on BMI, WHR or height of previous GIANT analyses. In the plots the p-values of the first stage meta analyses are presented.

(1) avPC1

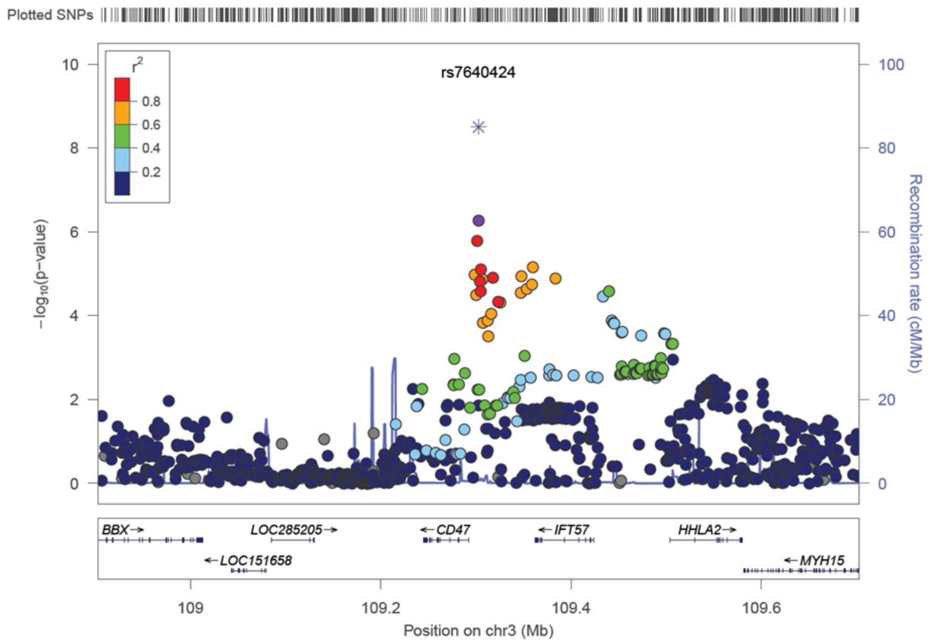

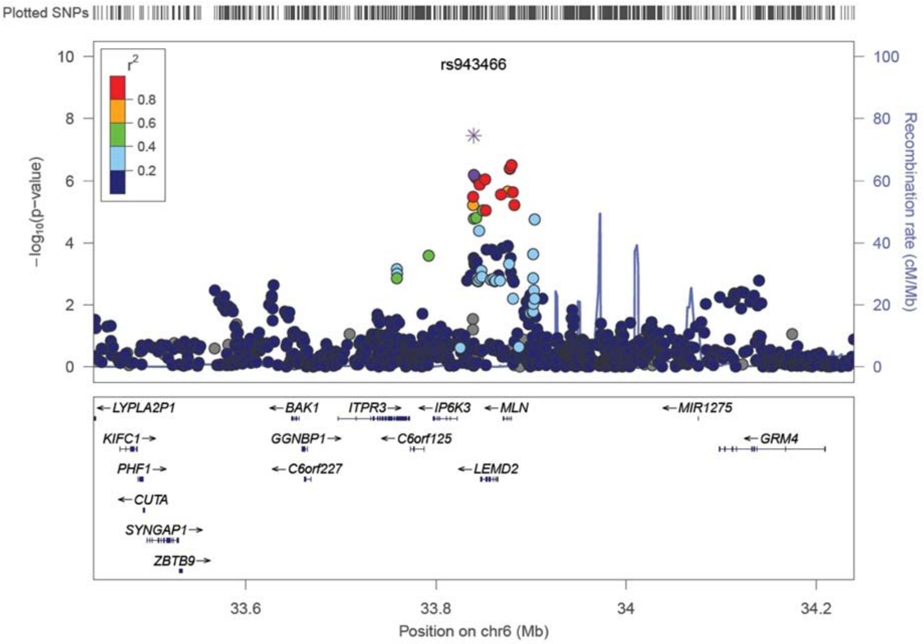

Plotted SNPs
rs943466
r2
0.8
0.6
0.4
0.2
−log10(p−value)
Recombination rate (cM/Mb)
LYPLA2P1
KIFC1
PHF1
CUTA
SYNGAP1
ZBTB9
BAK1
GGNBP1
C6orf227
ITPR3
IP6K3
C6orf125
MLN
LEMD2
MIR1275
GRM4
Position on chr6 (Mb)

(2) avPC3

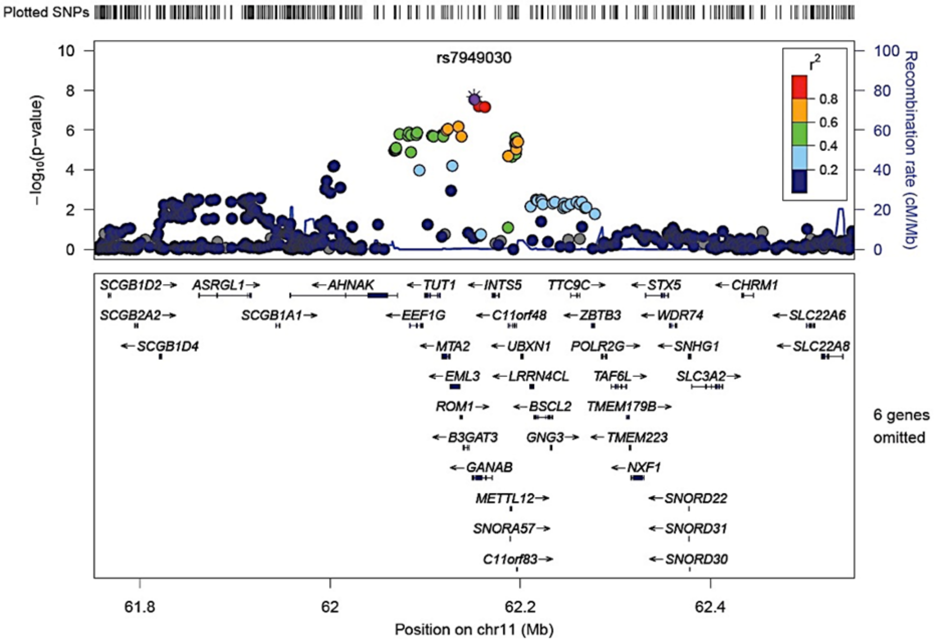

Plotted SNPs
rs7949030
r2
0.8
0.6
0.4
0.2
−log10(p−value)
Recombination rate (cM/Mb)
SCGB1D2
SCGB2A2
SCGB1D4
ASRGL1
SCGB1A1
AHNAK
TUT1
EEF1G
MTA2
EML3
ROM1
B3GAT3
GANAB
INTS5
C11orf48
UBXN1
LRRN4CL
BSCL2
GNG3
METTL12
SNORA57
C11orf83
TTC9C
ZBTB3
POLR2G
TAF6L
TMEM179B
TMEM223
NXF1
STX5
WDR74
SNHG1
SLC3A2
SNORD22
SNORD31
SNORD30
CHRM1
SLC22A6
SLC22A8
6 genes omitted
Position on chr11 (Mb)

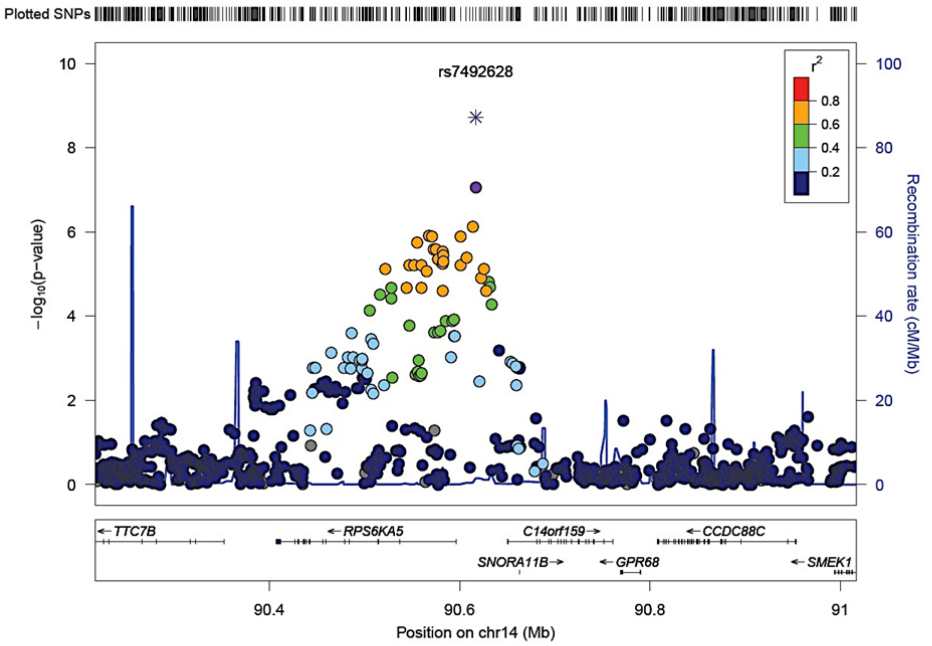
Plotted SNPs
rs7492628
r²
0.8
0.6
0.4
0.2
−log10(p−value)
Recombination rate (cM/Mb)
TTC7B
RPS6KA5
C14orf159
CCDC88C
SNORA11B
GPR68
SMEK1
90.4
90.6
90.8
91
Position on chr14 (Mb)

45

(4) avPC4

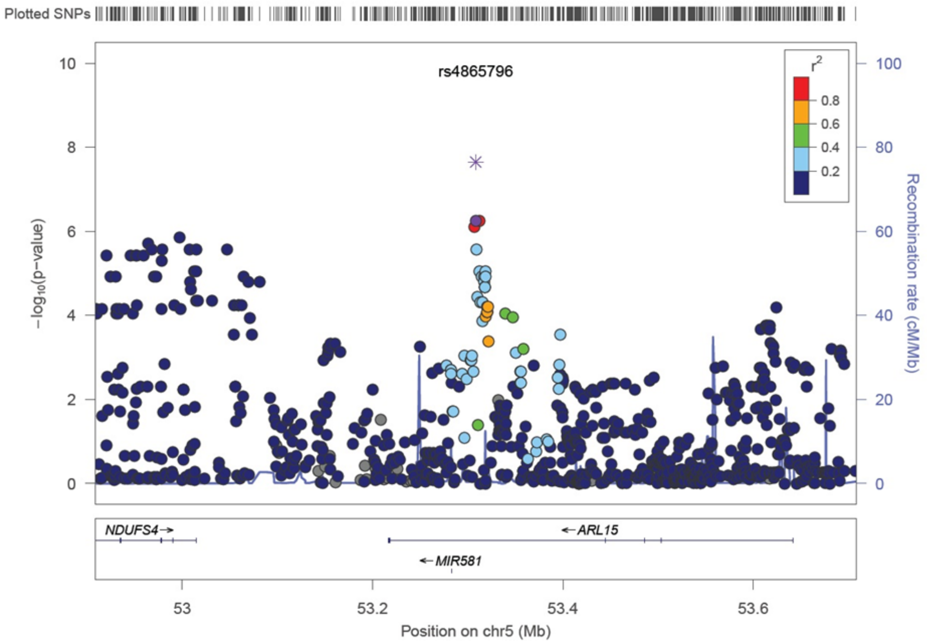
Plotted SNPs
rs4865796
r²
0.8
0.6
0.4
0.2
−log10(p-value)
Recombination rate (cM/Mb)
NDUFS4→
←ARL15
←MIR581
53
53.2
53.4
53.6
Position on chr5 (Mb)

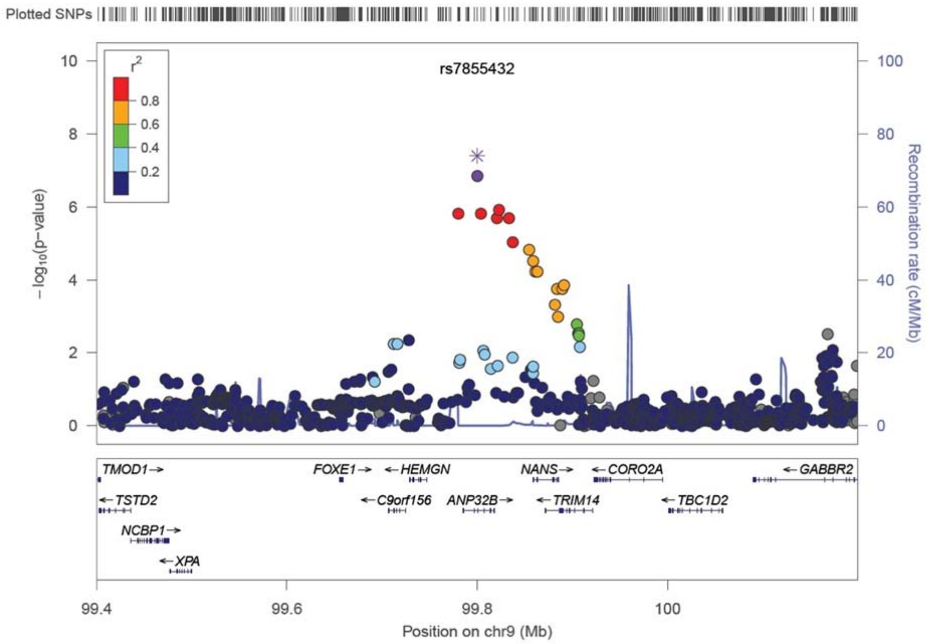
Plotted SNPs
rs7855432
r²
0.8
0.6
0.4
0.2
−log10(p-value)
Recombination rate (cM/Mb)
TMOD1→
←TSTD2
NCBP1→
←XPA
FOXE1→
←HEMGN
←C9orf156
ANP32B→
NANS→
←TRIM14
←CORO2A
←TBC1D2
←GABBR2
99.4
99.6
99.8
100
Position on chr9 (Mb)

Supplementary Figure 6: Venn Diagram of known anthropometric loci in body shape results. The Venn Diagrams report the overlap of loci that were reported in the latest GIANT meta analyses on one trait of BMI, height and WHR or for any of those traits (all traits) and identified for an avPC. In the upper right corner of each diagram the number of loci associated with the trait is given that were not identified for any avPC. A locus is regarded to the same if the lead SNPs are in LD > 0.8 and less than 500kb away from each other.

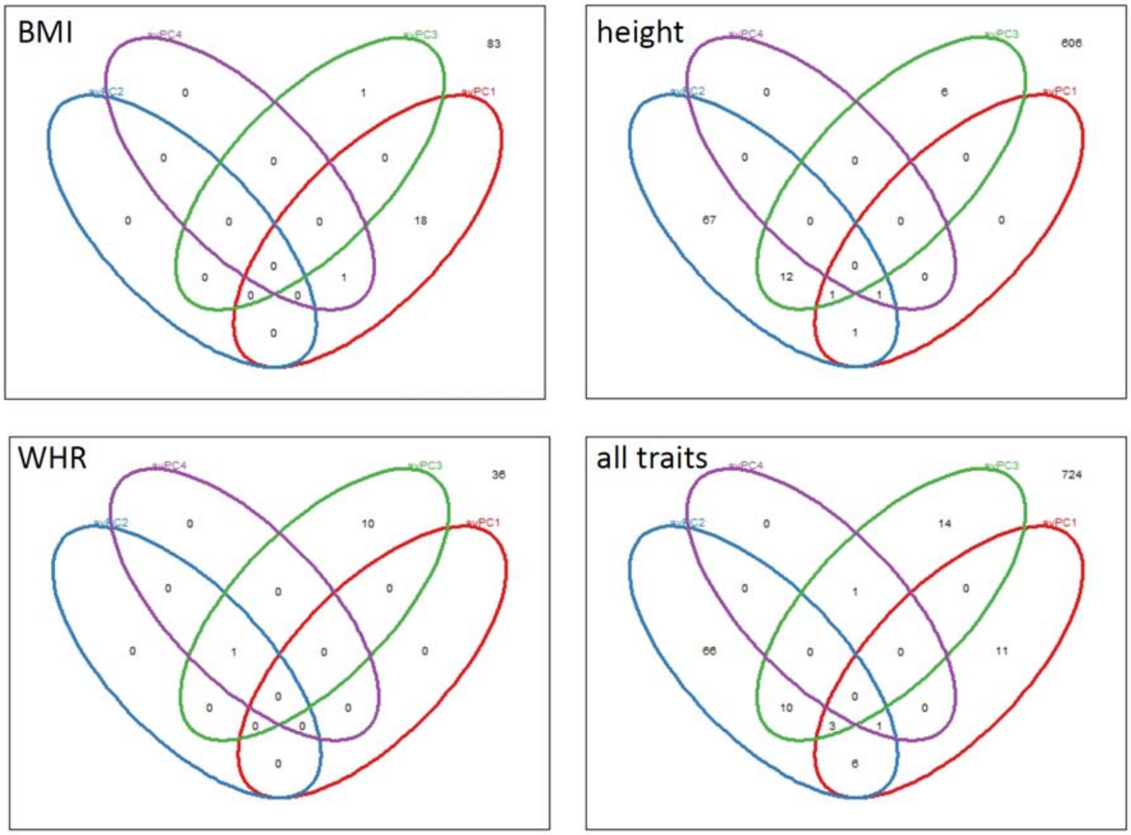

Supplementary Figure 7: Loci that were genome-wide significant for more than one avPC.

This Venn diagram shows which loci were significantly associated with more than one avPC. Loci of two avPCs are considered to overlap if the best genome-wide significant SNPs per locus are in strong LD ($R^2 > 0.8$).

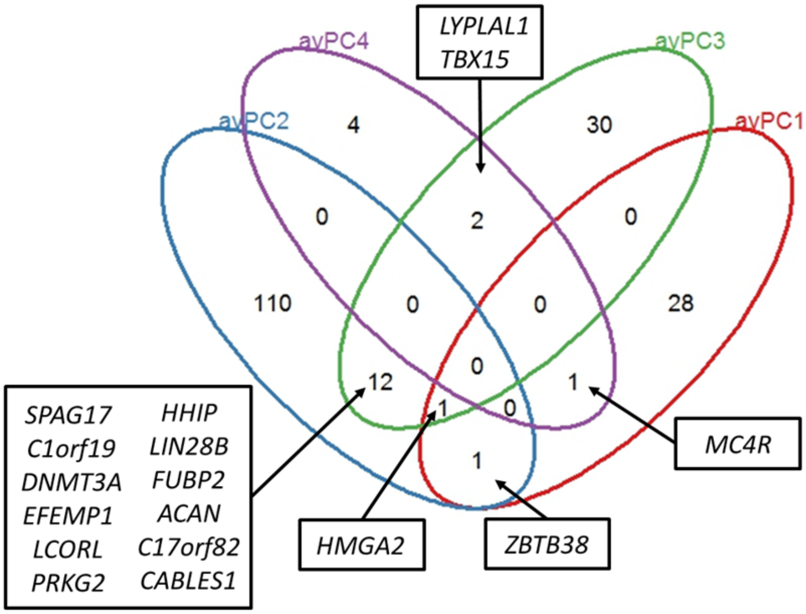

Supplement Tables:

Supplementary Table 1: Studies analysed in the pilot study. All studies are population based and are unrelated.

Study	N
ARIC	9,713
CROATIA_KORCULA	530
CROATIA_VIS	518
D2D2004	2,429
D2D2007	2,711
deCODE	4,779
DRSEXTRA	1,408
EGCUT	35,125
EPIC	2,390
Fenland	1,402
FHS	1,659
KORAF3	1,605
KORAS4	1,813
METSIM	0
ORCADES	312
RS-I	5,974
RS-II	1,911
RS-III	1,927
SardiNIA	887
SAVITAIPALE	1,194
SHIP	4,068
total	82,355

Supplementary Table 2: Loadings and explained variance of the avPCs. The average loadings that are used for calculation of the avPVs are given in this table. For each avPC the average of explained variance is given in this table.

	avPC1	avPC2	avPC3	avPC4	avPC5	avPC6
BMI	-0.473	-0.128	-0.284	0.504	0.635	-0.148
Height	-0.131	0.803	0.513	0.018	0.265	-0.062
Hip	-0.444	0.197	-0.399	-0.583	0.110	0.503
Waist	-0.488	-0.159	0.138	-0.424	-0.163	-0.715
Weight	-0.486	0.186	-0.056	0.475	-0.690	0.155
WHR	-0.297	-0.490	0.689	-0.019	0.105	0.432
Explained variance	64.37%	18.46%	13.79%	2.97%	0.26%	0.15%
Explained variance avPC1-avPC4	99.59%					

Supplementary Table 3: Number of studies and individuals per analysis stage. For each stage the maximal number of individuals is given that are used for analysis on body shape avPCs. In the second stage studies with genome wide data are analysed as well as studies with Metabochip data (N).

	Number of studies	Number of individuals
1st stage	43	133,376
2nd stage	22 (metabochip =12)	39,904 (metabochip= 32,170)
1st + 2nd combined	65	173,278

Supplementary Table 4: Summary of genome-wide significant, promising and novel loci.

	Promising Loci	Genome-Wide Significant	Novel
avPC1	56	31	2
avPC2	205	124	0
avPC3	89	45	2
avPC4	35	7	2
	385	207	6

Supplementary Table 5: Loci that were genome wide significant for more than one avPC.

Supplementary Table 5a: Loci that were genome wide significant for two avPCs. Loci of two avPCs are considered to be the same if the best genome wide significant SNPs per locus are in LD > 0.8. (Genome wide significant as defined in the text: promising p-value in the first stage meta analysis (<5x10-6) and genome wide significant in first and second stage combined analysis (<5x10-8).)

avPCs	snp1	snp2	chr	pos. snp1	pos. snp2	distance	LD	next gene
avPC1,avPC2	rs1582874	rs724016	3	142,597,909	142,588,260	9,649	1.00	*ZBTB38*
avPC1,avPC4	rs6567160	rs476828	18	55,980,115	56,003,567	23,452	1.00	*MC4R*
avPC3,avPC4	rs10923724	rs10923712	1	119,348,365	119,306,957	41,408	0.86	*TBX15*
avPC3,avPC4	rs2791550	rs2605100	1	217,721,992	217,710,847	11,145	0.94	*LYPLAL1*
avPC2,avPC3	rs7536458	rs7536458	1	118,666,125	118,666,125	0	1.00	*SPAG17*
avPC2,avPC3	rs1046934	rs2274432	1	182,290,152	182,287,568	2,584	1.00	*C1orf19*
avPC2,avPC3	rs2289195	rs2289195	2	25,316,987	25,316,987	0		
avPC2,avPC3	rs3791675	rs3791675	2	55,964,813	55,964,813	0	1.00	*EFEMP1*
avPC2,avPC3	rs6853216	rs4057984	4	17,579,753	17,566,298	13,455	1.00	*LCORL*
avPC2,avPC3	rs1975474	rs1115919	4	82,397,961	82,392,421	5,540	0.86	*PRKG2*
avPC2,avPC3	rs7689420	rs7689420	4	145,787,802	145,787,802	0	1.00	*HHIP*
avPC2,avPC3	rs314263	rs7759938	6	105,499,438	105,485,647	13,791	0.95	*LIN28B*
avPC2,avPC3	rs7466269	rs7021911	9	132,453,905	132,490,839	36,934	0.91	*FUBP3*

avPC2,avPC3	rs2280470	rs2280470	15	87,196,630	87,196,630	0	1.00	*ACAN*
avPC2,avPC3	rs2079795	rs9892365	17	56,851,431	56,846,166	5,265	1.00	*C17orf82*
avPC2,avPC3	rs4239437	rs4239436	18	18,986,222	18,985,928	294	1.00	*CABLES1*

Supplementary Table 5b: Loci that were genome wide significant for three avPCs. Loci of two avPCs are considered to be the same if the best genome wide significant SNPs per locus are in LD > 0.9. (Genome wide significant as defined in the text: promising p-value in the first stage meta analysis (<5x10-6) and genome wide significant in first and second stage combined analysis (<5x10-8).)

**avPCs**	**snp1**	**snp2**	**snp3**	**chr**	**pos. SNP1/SNP2/SNP3**	**distance (snp1-2/snp1-3/snp2-3)**	**LD (snp1-2/snp1-3/snp2-3)**	**next gene**
avPC1,avPC2, avPC3	rs7970350	rs1351394	rs8756	12	64,646,431/ 64,638,093/ 64,646,019	8338/412/7926	0.91/0.89/0.97	*HMGA2*

Supplement Notes:

AUTHOR CONTRIBUTIONS

Ruth J.F. Loos, Martina Müller-Nurasyid lead the steering committee and oversaw the consortium. The writing group consisted of Janina S Ried, Janina Jeff, Audrey Y Chu, Jennifer L Bragg-Gresham, Jenny van Dongen, Jennifer E Huffman, Martina Müller-Nurasyid, and Ruth JF Loos. The method development for the PCA approach was conducted by Janina S Ried, Martina Müller-Nurasyid. Data cleaning and preparation was performed by Janina S Ried, Jenny van Dongen, and Jennifer E Huffman. The GWAS and Metabochip Meta-analyses and follow-up analyses were carriedout by Janina S Ried, Janina Jeff, Audrey Y Chu, Jennifer L Bragg-Gresham, Jenny van Dongen, and Jennifer E Huffman.

The data analysis was a collective effort by Tarunveer Singh Ahluwalia, Eva Albrecht, Traci M Bartz, John Blangero, Jennifer L Bragg-Gresham, Gemma Cadby, Daniel I. Chasman, Charleston W.K. Chiang, L Adrienne Cupples, Niina Eklund, Joel Eriksson, Tõnu Esko, Teresa Ferreira, Krista Fischer, Anuj Goel, Mathias Gorski, Mariaelisa Graff, Caroline Hayward, Nancy L Heard-Costa, Frank Hu, Jennifer E Huffman, David Hunter, Aaron Isaacs, Anne U Jackson,

Janina Jeff, Anne E Justice, Stavroula Kanoni, Robert C Kaplan, Kati Kristiansson, Zoltán Kutalik, Jari Lahti, Terho Lehtimäki, Mattias Lorentzon, Jian'an Luan, Anubha Mahajan, Massimo Mangino, Irene Mateo Leach, Barbara McKnight, Carolina Medina-Gomez, Evelin Mihailov, Keri L Monda, May E Montasser, Andrew P Morris, Gabriele Müller, Martina Müller-Nurasyid, Ilja M Nolte, Jeffrey R O'Connell, Claes Ohlsson, Ben A. Oostra, Louis Pérusse, Lu Qi, Olli T Raitakari, Nigel W Rayner, Janina S. Ried, Fernando Rivadeneira, Lynda M. Rose, Kathy A Ryan, Erika Salvi, Serena Sanna, Megan T Smith, Harold Snieder, Lorraine Southam, Thomas H Sparso, Alena Stančáková, Valgerdur Steinthorsdottir, David P Strachan, Ioanna Tachmazido, Alexander Teumer, Gudmar Thorleifsson, Pim van der Harst, Jana V Van Vliet-Ostaptchouk, Liesbeth Vandenput, Niek Verweij, Veronique Vitart, Sophie R. Wang, Thomas W Winkler, Andrew Wong, Laura M Yerges-Armstrong, and Weihua Zhang.

Project design, management and coordination of contributing studies was lead by the following co-authors: Goncalo R Abecasis, John Beilby, Sven Bergmann, Michael Boehnke, Stefan R Bornstein, Harry Campbell, John C Chambers, Francis S Collins, Fracensco Cucca, L Adrienne Cupples, Daniele Cusi, Panos Deloukas, Martin Farrall, Nita G Forouhi, Caroline S Fox, Ron T Ganzevoort, Christian Gieger, Anders Hamsten, Torben Hansen, Nicholas Hastie, Caroline Hayward, Markku Heliövaara, Andrew A Hicks, Joel N.

Hirschhorn, Albert Hofman, Frank Hu, David Hunter, Lise LotteHusemoen, Kristian Hveem, Alan L James, Marjo-Riitta Jarvelin, Torben Jorgensen, Pekka Jousilahti, Antti Jula, Mika Kähönen, Eero Kajantie, Paul B Knekt, Heikki A Koistinen, Jaspal S Kooner, Seppo Koskinen, Diana Kuh, Johanna Kuusisto, Markku Laakso, Timo A Lakka, Claudia Langenberg, Terho Lehtimäki, Cecilia M Lindgren, Allan Linneberg, Ruth J.F. Loos, Mark I McCarthy, Andres Metspalu, Arthur W Musk, Inger Njølstad, Kari E North, Claes Ohlsson, Albertine J Oldehinkel, Ken K Ong, Ben A. Oostra, Clive Osmond, Lyle J Palmer, Oluf B Pedersen, Markus Perola, Louis Pérusse, Annette Peters, Ozren Polašek, Peter P Pramstaller, Bruce M. Psaty, Hannu Puolijoki, Lu Qi, Olli T Raitakari, Rainer Rauramaa, Paul M Ridker, Fernando Rivadeneira, Igor Rudan, Veikko Salomaa, David Schlessinger, Peter EH Schwarz, Robert A Scott, Alan R Shudiner, Harold Snieder, Thorkild IA Sorensen, Tim D Spector, Kari Stefansson, David P Strachan, Konstantin Strauch, Michael Stumvoll, Unnur Thorsteinsdottir, Angelo Tremblay, Jaakko Tuomilehto, André G. Uitterlinden, Matti Uusitupa, Pim van der Harst, Cornelia M. van Duijn, Erkki Vartiainen, Jorma S Viikari, Veronique Vitart, Marie-Claude Vohl, Peter Vollenweider, Gérard Waeber, Nicholas J Wareham, Hugh Watkins, Sarah Wild, James F Wilson, Alan F Wright, and Eleftheria Zeggini.

Genotyping of contributing studies was performed by Cristina Barlassina, John Beilby, Claire Bellis, John Blangero, Lori L Bonnycastle, Marcel Bruinenberg, Harry Campbell, John C Chambers, Daniel I. Chasman, Yii-Der Ida Chen, Peter S Chines, Francesca D'Avila, Maria Dimitriou, Tõnu Esko, Nele Friedrich, Anette Prior Gjesing, Harald Grallert, Niels Grarup, Marie Neergaard Harder, Catharina A Hartman, Andrew Tym Hattersley, Caroline Hayward, Andrew A Hicks, Georg Homuth, Frank Hu, Jennie Hui, David Hunter, Pirro G Hysi, Shapour Jalilzadeh, Marjo-Riitta Jarvelin, Johanne M Justesen, Leena Kinnunen, Jaspal S Kooner, Peter Kovacs, Diana Kuh, Theodosios Kyriakou, Claudia Langenberg, Terho Lehtimäki, Mattias Lorentzon, Wendy L McArdle, Mark I McCarthy, Carolina Medina-Gomez, Lili Milani, Narisu Narisu, Kari E North, Jeffrey R O'Connell, Claes Ohlsson, Ken K Ong, Ben A. Oostra, Aarno Palotie, Lavinia Paternoster, Lu Qi, Nigel W Rayner, Rasmus Ribel-Madsen, Marcus Richards, Fernando Rivadeneira, Lynda M. Rose, Sylvain Sebert , Lorraine Southam, Kathleen Stirrups, Michael Stumvoll, Morris A Swertz, Amy J Swift, Ioanna Tachmazido, Alexander Teumer, Anke Tönjes, André G. Uitterlinden, Pim van der Harst, Cornelia M. van Duijn, Cristina Venturini, Veronique Vitart, Uwe Völker, Nicholas J Wareham, Elisabeth Widén, James F Wilson, and Andrew Wong.

Phenotype coordination of contributing studies was a collective effort by the following: Alexessander Couto Alves, Stephan JL Bakker, Matthias Blüher, John C Chambers, Daniel I. Chasman, Daniele Cusi, George Dedoussis, Joel Eriksson, Aliki-Eleni Farmaki, Nita G Forouhi, Caroline S Fox, Ron T Ganzevoort, Nicola Glorioso, Jürgen Gräßler, Jagvir Grewal, Catharina A Hartman, Maija Hassinen, Andrew Tym Hattersley, Aki S Havulinna, Iris M Heid, Hans Hillege, Oddgeir Holmen, Frank Hu, David Hunter, Lise Lotte Husemoen, Pirro G Hysi, Till Ittermann, Alan L James, Eero Jokinen, Torben Jorgensen, Mika Kähönen, Maria Karaleftheri, Ivana Kolcic, Ishminder K Kooner, Jaspal S Kooner, Peter Kovacs, Diana Kuh, Jari Lahti, Tomi Laitinen, Alexandra M. Lewin, Peter Lichtner, Jaana Lindström, Allan Linneberg, Roberto Lorbeer, Mattias Lorentzon, Reedik Mägi, Massimo Mangino, Satu Männistö, Paolo Manunta, Carolina Medina-Gomez, Andres Metspalu, Rebecca Mills, Gabriele Müller, Arthur W Musk, Kari E North, Claes Ohlsson, Lyle J Palmer, Annette Peters, Irene Pichler, Maria G Pilia, Ozren Polašek, Inga Prokopenko, Bruce M. Psaty, Lu Qi, Olli T Raitakari, Nigel W Rayner, Marcus Richards, Fernando Rivadeneira, Lynda M. Rose, Igor Rudan, Veikko Salomaa, Salome Scholtens, Alan R Shudiner, Thorkild I. A. Sorensen, Ronald P Stolk, David P Strachan, Heather M Stringham, Morris A Swertz, Anke Tönjes, Angelo Tremblay, Emmanouil Tsafantakis, André G. Uitterlinden, Peter J Van der Most, Liesbeth Vandenput,

Jorma S Viikari, Judith M Vonk, Nicholas J Wareham, Sarah Wild, Tom Wilsgaard, James F Wilson, and Carola Zillikens.

We have the following disclosures to declare: Kari Stefansson, Valgerdur Steinthorsdottir, Gudmar Thorleifsson, and Unnur Thorsteinsdottir are employed by deCODE Genetics/Amgen inc. Gérard Waeber and Peter Vollenweider received an unrestricted grant from GSK to build the CoLaus study. Bruce M. Psaty serves on a DSMB for a clinical trial of a device funded by the manufacturer (Zoll LifeCor)

Acknowledgements

Amish, we gratefully acknowledge our Amish liaisons, field workers and clinic staff and the extraordinary cooperation and support of the Amish community without which these studies would not have been possible. The Amish studies are supported by grants and contracts from the NIH, including U01 HL072515-06, U01 HL84756, U01HL105198, U01 GM074518, F32AR059469, the University of Maryland General Clinical Research Center, grant M01 RR 16500, by the T32 training grant AG000219, and by National Research Initiative Competitive Grant no. 2007-35205-17883 from the USDA National Institute of Food and Agriculture. We thank our Amish research volunteers for their long-

standing partnership in research, and the research staff at the Amish Research Clinic for their hard work and dedication.

ARIC, the Atherosclerosis Risk in Communities Study is carried out as a collaborative study supported by National Heart, Lung, and Blood Institute contracts N01-HC-55015, N01-HC-55016, N01-HC-55018, N01-HC-55019, N01-HC-55020, N01-HC-55021, N01-HC-55022, R01HL087641, R01HL59367 and R01HL086694; National Human Genome Research Institute contract U01HG004402; and National Institutes of Health contract HHSN268200625226C. Infrastructure was partly supported by Grant Number UL1RR025005, a component of the National Institutes of Health and NIH Roadmap for Medical Research. The project described was supported by Grant Number UL1 RR 025005 from the National Center for Research Resources (NCRR), a component of the National Institutes of Health (NIH) and NIH Roadmap for Medical Research, and its contents are solely the responsibility of the authors and do not necessarily represent the official view of NCRR or NIH. The authors thank the staff and participants of the ARIC Study for their important contributions.

British 1958 Birth Cohort (B58C), data collection was funded by MRC grant G0000934 and cell-line creation by Wellcome Trust grant 068545/Z/02. Great Ormond Street Hospital/University College London, Institute of Child Health and Oxford Biomedical Research Centre, University of Oxford received a proportion of funding from the Department of Health's National Institute for Health Research (NIHR) ('Biomedical Research Centres' funding).

This paper presents independent research and the views expressed are those of the author(s) and not necessarily those of the NHS, the NIHR, or the Department of Health."

The Busselton Health Study,BHS/BSN, acknowledges the generous support for the 1994/5 follow-up study from Healthway, Western Australia and the numerous Busselton community volunteers who assisted with data collection and the study participants from the Shire of Busselton. The Busselton Health Study is supported by The Great Wine Estates of the Margaret River region of Western Australia. The BHS gratefully acknowledges the assistance of the Western Australian DNA Bank (NHMRC Enabling Facility) with DNA samples and the support provided by the Ark (NHMRC Enabling Facility) for this study.

CHS, the CHS research was supported by NHLBI contracts HHSN268201200036C, HHSN268200800007C, N01HC55222, N01HC85079, N01HC85080, N01HC85081, N01HC85082, N01HC85083, N01HC85086; and NHLBI grants U01HL080295, R01HL087652, R01HL105756, R01DK089256, R01HL103612, and R01HL120393 with additional contribution from the National Institute of Neurological Disorders and Stroke (NINDS). Additional support was provided through R01AG023629 from the National Institute on Aging (NIA). A full list of principal CHS investigators and institutions can be found at CHS-NHLBI.org.The provision of genotyping data was supported in part by the National Center for Advancing Translational Sciences, CTSI grant UL1TR000124, and the National Institute of Diabetes and Digestive and Kidney Disease Diabetes Research Center (DRC) grant DK063491 to the Southern California Diabetes Endocrinology Research Center. All CHS participants

included in these analyses gave written informed consent, including consent to participate in genetic studies.

COLAUS, the CoLaus study was supported by research grants from the Swiss National Science Foundation (grant no: 33CSCO-122661, 33CS30-139468 and 33CS30-148401) fromGlaxoSmithKline and the Faculty of Biology and Medicine of Lausanne, Switzerland. The authors also express their gratitude to the participants in the Lausanne CoLaus study and to the investigators who have contributed to the study in particular Vincent Mooser and Dawn Waterworth, and the research nurses for data collection. ZK received financial support from Swiss National Science Foundation (grant no: 31003A-143914) and the Leenaards Foundation.

COROGENE/FINRISK, this study has been funded by the Academy of Finland (grant numbers 139635, 129494, 118065, 129322, 250207, 136895, 263836), the Orion-Farmos Research Foundation, the Finnish Foundation for Cardiovascular Research, and the Sigrid Jusélius Foundation. The COROGENE controls are part of the national FINRISK study. We are grateful for the THL DNA laboratory for its skillful work to produce the DNA samples used in this study. We thank the Sanger Institute genotyping facilities for genotyping the samples.

CROATIA_KORCULA, the CROATIA-Korcula study was funded by grants from the Medical Research Council (UK), European Commission Framework 6 project EUROSPAN (Contract No. LSHG-CT-2006-018947), the Republic of Croatia Ministry of Science, Education and Sports research grants (216-1080315-0302) and the Croatian Science Foundation (grant 8875). We would

like to acknowledge the invaluable contributions of the recruitment team in Korcula, the administrative teams in Croatia and Edinburgh and the people of Korcula. The SNP genotyping for the CROATIA-Korcula cohort was performed in Helmholtz Zentrum München, Neuherberg, Germany.

CROATIA_SPLIT, the CROATIA-Split and CROATIA-Korcula studies were funded by grants from the Medical Research Council (UK), European Commission Framework 6 project EUROSPAN (Contract No. LSHG-CT-2006-018947) and Republic of Croatia Ministry of Science, Education and Sports research grants to I.R. (108-1080315-0302). We would like to acknowledge the staff of several institutions in Croatia that supported the field work, including but not limited to The University of Split and Zagreb Medical Schools and the Croatian Institute for Public Health. The SNP genotyping for the CROATIA-Split cohort was performed by AROS Applied Biotechnology, Aarhus, Denmark.

CROATIA_VIS, the CROATIA-Vis study was funded by grants from the Medical Research Council (UK) and Republic of Croatia MinistryThe CROATIA-Vis study was funded by grants from the Medical Research Council (UK) and Republic of Croatia Ministry of Science, Education and Sports research grants to I.R. (108-1080315-0302). We would like to acknowledge the staff of several institutions in Croatia that supported the field work, including but not limited to The University of Split and Zagreb Medical Schools, the Institute for Anthropological Research in Zagreb and Croatian Institute for Public Health. The SNP genotyping for the CROATIA-Vis cohort was performed in the core genotyping laboratory of the Wellcome Trust Clinical Research Facility at the Western General Hospital, Edinburgh, Scotland of Science, Education and Sports research grants to I.R. (108-1080315-0302). We would like to

acknowledge the staff of several institutions in Croatia that supported the field work, including but not limited to The University of Split and Zagreb Medical Schools, the Institute for Anthropological Research in Zagreb and Croatian Institute for Public Health.

The deCODE consortium thanks participants in deCODE cardiovascular- and obesity studies and collaborators for their cooperation. The research performed at deCODE Genetics was part funded through the European Community's Seventh Framework Programme (FP7/2007-2013), ENGAGE project, grant agreement HEALTH-F4-2007- 201413.

Diabetes Genetics Initiative (DGI), the Botnia (DGI) study have been supported by grants from Folkhälsan Research Foundation, Sigrid Juselius Foundation, Ministry of Education, Nordic Center of Excellence in Disease Genetics, Gyllenberg Foundation, Swedish Cultural Foundation in Finland, Finnish Diabetes Research Foundation, Foundation for Life and Health in Finland, Finnish Medical Society, Paavo Nurmi Foundation, Perklén Foundation, Ollqvist Foundation, Närpes Health Care Foundation, the Municipal Health Care Center and Hospital in Jakobstad, Health Care Centers in Vasa, Närpes and Korsholm. This work was also partially supported by NIH grant R01-DK075787 to JNH.

EPIC-Obesity Study (EPIC), the EPIC Norfolk diabetes case cohort study is nested within the EPIC Norfolk Study,which is supported by programme grants from the Medical Research Council, and Cancer Research UK and with additional support from the European Union, Stroke Association, British Heart Foundation, Research into Ageing, Department of Health, The Wellcome Trust and the Food Standards Agency. Genotyping was in part supported by the

MRC-GSK pilot programme grant. We acknowledge the contribution of the staff and participants of the EPIC-Norfolk Study.

Erasmus family study (ERF), the ERF study as a part of EUROSPAN (European Special Populations Research Network) was supported by European Commission FP6 STRP grant number 018947 (LSHG-CT-2006-01947) and also received funding from the European Community's Seventh Framework Programme (FP7/2007-2013)/grant agreement HEALTH-F4-2007-201413 by the European Commission under the programme "Quality of Life and Management of the Living Resources" of 5th Framework Programme (no. QLG2-CT-2002-01254). The ERF study was further supported by ENGAGE consortium and CMSB. High-throughput analysis of the ERF data was supported by joint grant from Netherlands Organisation for Scientific Research and the Russian Foundation for Basic Research (NWO-RFBR 047.017.043).ERF was further supported by the ZonMw grant (project 91111025). We are grateful to all study participants and their relatives, general practitioners and neurologists for their contributions and to P. Veraart for her help in genealogy, J. Vergeer for the supervision of the laboratory work and P. Snijders for his help in data collection.

Estonian Genome Project (EGCUT), this work was supported by the Targeted Financing from the Estonian Ministry of Science and Education [SF0180142s08]; the US National Institute of Health [R01DK075787]; the Development Fund of the University of Tartu (grant SP1GVARENG); the European Regional Development Fund to the Centre of Excellence in Genomics (EXCEGEN; grant 3.2.0304.11-0312); and through FP7 grant 313010.

Family Heart Study (FamHS) was supported by the following grants 1R01DK8925601 and 5R01DK075681 (IBB)

Fenland, the Fenland Study is funded by the Wellcome Trust and the Medical Research Council (MC_U106179471). We are grateful to all the volunteers for their time and help, and to the General Practitioners and practice staff for assistance with recruitment. We thank the Fenland Study Investigators, Fenland Study Co-ordination team and the Epidemiology Field, Data and Laboratory teams.

Framingham Heart Study (FHS, FRAM), this research was conducted in part using data and resources from the Framingham Heart Study of the National Heart Lung and Blood Institute of the National Institutes of Health and Boston University School of Medicine. The analyses reflect intellectual input and resource development from the Framingham Heart Study investigators participating in the SNP Health Association Resource (SHARe) project. This work was partially supported by the National Heart, Lung and Blood Institute's Framingham Heart Study (Contract No. N01-HC-25195) and its contract with Affymetrix, Inc for genotyping services (Contract No. N02-HL-6-4278). A portion of this research utilized the Linux Cluster for Genetic Analysis (LinGA-II) funded by the Robert Dawson Evans Endowment of the Department of Medicine at Boston University School of Medicine and Boston Medical Center.

FUSION, the FUSION GWAS cases and controls Support for FUSION was provided by NIH grants R01-DK062370 (to M.B.), R01-DK072193 (to K.L.M.), and intramural project number 1Z01-HG000024 (to F.S.C.). Genome-wide genotyping was conducted by the Johns Hopkins University Genetic Resources

Core Facility SNP Center at the Center for Inherited Disease Research (CIDR), with support from CIDR NIH contract no. N01-HG-65403.

GENMETS/H2000 (H2000_case + H2000_control), H2000, would like to thank all study participants. The Health 2000 Study was funded by the National Institute for Health and Welfare (THL), the Finnish Centre for Pensions (ETK), the Social Insurance Institution of Finland (KELA), the Local Government Pensions Institution (KEVA) and other organizations listed on the website of the survey (http://www.terveys2000.fi). We are grateful for the THL DNA laboratory for its skillful work to produce the DNA samples used in this study. We thank the Sanger Institute genotyping facilities for genotyping the GenMets subcohort.

GOOD GOOD financial support was received from the Swedish Research Council, the Swedish Foundation for Strategic Research, the ALF/LUA research grant in Gothenburg, the Lundberg Foundation, the Torsten and Ragnar Söderberg's Foundation, the Novo Nordisk Foundation, and the European Commission grant HEALTH-F2-2008-201865-GEFOS.

HBCS would like to thank all study participants as well as everybody involved in the Helsinki Birth Cohort Study. Helsinki Birth Cohort Study has been supported by grants from the Academy of Finland (130326, 134791, 263924), the Finnish Diabetes Research Society, Folkhälsan Research Foundation, Novo Nordisk Foundation, Finska Läkaresällskapet, Signe and Ane Gyllenberg Foundation, Ahokas Foundation, Emil Aaltonen Foundation, Juho Vainio Foundation, Finnish Foundation for Pediatric Research, Sigrid Juselius Foundation, the Finnish Special Governmental Subsidy for Health Sciences,

Samfundet Folkhälsan, Liv och Hälsa, and Wellcome Trust (grant number WT089062). We are grateful for the THL DNA laboratory for its skillful work to produce the DNA samples used in this study. We thank the Sanger Institute genotyping facilities for genotyping the samples.

HYPERGENES, Hypergenes is made possible with funding by HYPERGENES (FP7 - HEALTH-F4-2007-201550); INTEROMICS (MIUR - CNR Italian Flagship Project); InGenious HyperCare (LSHM-CT-2006-037093)

KORA_F3 + KORA F4, the KORA research platform (KORA, Cooperative Health Research in the Region of Augsburg) was initiated and financed by the Helmholtz Zentrum München - German Research Center for Environmental Health, which is funded by the German Federal Ministry of Education and Research and by the State of Bavaria. Furthermore, KORA research was supported within the Munich Center of Health Sciences (MC Health), Ludwig-Maximilians-Universität, as part of LMUinnovativ.

Lifelines, the LifeLines Cohort Study, and generation and management of GWAS genotype data for the LifeLines Cohort Study is supported by the Netherlands Organization of Scientific Research NWO (grant 175.010.2007.006), the Economic Structure Enhancing Fund (FES) of the Dutch government, the Ministry of Economic Affairs, the Ministry of Education, Culture and Science, the Ministry for Health, Welfare and Sports, the Northern Netherlands Collaboration of Provinces (SNN), the Province of Groningen, University Medical Center Groningen, the University of Groningen, Dutch Kidney Foundation and Dutch Diabetes Research Foundation. We thank Behrooz Alizadeh, Annemieke Boesjes, Marcel Bruinenberg, Noortje Festen,

Pim van der Harst, Ilja Nolte, Lude Franke, Mitra Valimohammadi for their help in creating the GWAS database, and Rob Bieringa, Joost Keers, René Oostergo, Rosalie Visser, Judith Vonk for their work related to data-collection and validation. The authors are grateful to the study participants, the staff from the LifeLines Cohort Study and the contributing research centers delivering data to LifeLines and the participating general practitioners and pharmacists.

MICROS, the MICROS study, would like to thank the primary care practitioners Raffaela Stocker, Stefan Waldner, Toni Pizzecco, Josef Plangger, Ugo Marcadent, and the personnel of the Hospital of Silandro (Department of Laboratory Medicine) for their participation and collaboration in the research project.The MICROS study was supported by the Ministry of Health and Department for Promotion of Educational Policies, Universities and Research of the Autonomous Province of Bolzano, South Tyrol, the South Tyrolean Sparkasse Foundation, and the European Union framework program 6 EUROSPAN project (contract no. LSHG-CT-2006-018947).

NESDA/NTR NTR-NESDA, the NTR study would like to thank all of our study participants for their continous voluntary contributions to our scientific efforts as well as the SURF SARA institute for their computational resources. Funding was obtained from the Netherlands Organization for Scientific Research (NWO) and The Netherlands Organisation for Health Research and Development (ZonMW) grants 904-61-090, 985-10-002, 912-10-020, 904-61-193,480-04-004, 463-06-001, 451-04-034, 400-05-717, Addiction-31160008, Middelgroot-911-09-032, Spinozapremie 56-464-14192, Biobanking and Biomolecular Resources Research Infrastructure (BBMRI –NL, 184.021.007). VU Institute for Health and Care Research (EMGO+); the European Community's Seventh Framework

Program (FP7/2007-2013), ENGAGE (HEALTH-F4-2007-201413); the European Research Council (ERC Advanced, 230374, ERC Starting grant 284167), Rutgers University Cell and DNA Repository (NIMH U24 MH068457-06), the Avera Institute, Sioux Falls, South Dakota (USA) and the National Institutes of Health (NIH, R01D0042157-01A, MH081802; R01 DK092127-04, Grand Opportunity grants 1RC2 MH089951 and 1RC2 MH089995). Part of the genotyping and analyses were funded by the Genetic Association Information Network (GAIN) of the Foundation for the National Institutes of Health. Computing was supported by BiG Grid, the Dutch e-Science Grid, which is financially supported by NWO.

NFBC66, funding NFBC1966 received financial support from University of Oulu Grant no. 65354, Oulu University Hospital Grant no. 2/97, 8/97, Ministry of Health and Social Affairs Grant no. 23/251/97, 160/97, 190/97, National Institute for Health and Welfare, Helsinki Grant no. 54121, Regional Institute of Occupational Health, Oulu, Finland Grant no. 50621, 54231.) . The DNA extractions, sample quality controls, biobank up-keeping and aliquotting was performed in the National Institute for Health and Welfare, Biomedicum Helsinki, Finland and supported financially by the Academy of Finland and Biocentrum Helsinki. We thank the late Professor Paula Rantakallio (launch of NFBC1966), and Dr Outi Törnwall and Ms Minttu Sauramo (DNA biobanking). The authors would like to acknowledge the contribution of the late Academian of Science Leena Peltonen, the participants in the 31yrs study and the NFBC project center. Reedik Mägi was funded by EU FP7 Marie Curie IEF fellowship. Sylvain Sebert was funded by the EU-H2020 DynaHEALTH [grant No 633595]

Nurses Health study (NHS) NHS NIH U01CA-098233, R01HL71981, DK091718, DK046200

ORCADES was supported by the Chief Scientist Office of the Scottish Government, the Royal Society, the MRC Human Genetics Unit, Arthritis Research UK and the European Union framework program 6 EUROSPAN project (contract no. LSHG-CT-2006-018947). DNA extractions were performed at the Wellcome Trust Clinical Research Facility in Edinburgh. We would like to acknowledge the invaluable contributions of Lorraine Anderson and the research nurses in Orkney, the administrative team in Edinburgh and the people of Orkney.

PREVEND genetics is supported by the Dutch Kidney Foundation (Grant E033), the EU project grant GENECURE (FP-6 LSHM CT 2006 037697), the National Institutes of Health (grant 2R01LM010098), The Netherlands organisation for health research and development (NWO-Groot grant 175.010.2007.006, NWO VENI grant 916.761.70, ZonMw grant 90.700.441), the Netherlands Heart Foundation (grant NHS2010B280) and the Dutch Inter University Cardiology Institute Netherlands (ICIN).

PROCARDIS was supported by the European Community Sixth Framework Program (LSHM-CT- 2007-037273), AstraZeneca, the British Heart Foundation, the Swedish Research Council, the Knut and Alice Wallenberg Foundation, the Swedish Heart-Lung Foundation, the Torsten and Ragnar Söderberg Foundation, the Strategic Cardiovascular Program of Karolinska Institutet and Stockholm County Council, the Foundation for Strategic Research and the Stockholm County Council (560283). M.F and H.W acknowledge the support of the Wellcome Trust core award (090532/Z/09/Z) and M.F, H.W and T.K, the BHF Centre of Research Excellence. A.G, H.W and T.K acknowledge European

Union Seventh Framework Programme FP7/2007-2013 under grant agreement no. HEALTH-F2-2013-601456 (CVGenes@Target) & and A.G, the Wellcome Trust Institutional strategic support fund.

RS-I, RS-II, and RS-III, the Rotterdam Study is funded by Erasmus Medical Center and Erasmus University, Rotterdam, Netherlands Organization for the Health Research and Development (ZonMw), the Research Institute for Diseases in the Elderly (RIDE), the Ministry of Education, Culture and Science, the Ministry for Health, Welfare and Sports, the European Commission (DG XII), and the Municipality of Rotterdam. The authors are grateful to the study participants, the staff from the Rotterdam Study and the participating general practitioners and pharmacists. The generation and management of GWAS genotype data for the Rotterdam Study (RS I, RS II, RS III) was executed by the Human Genotyping Facility of the Genetic Laboratory of the Department of Internal Medicine, Erasmus MC, Rotterdam, The Netherlands. The GWAS datasets are supported by the Netherlands Organisation of Scientific Research NWO Investments (nr. 175.010.2005.011, 911-03-012), the Genetic Laboratory of the Department of Internal Medicine, Erasmus MC, the Research Institute for Diseases in the Elderly (014-93-015; RIDE2), the Netherlands Genomics Initiative (NGI)/Netherlands Organisation for Scientific Research (NWO) Netherlands Consortium for Healthy Aging (NCHA), project nr. 050-060-810. We thank Pascal Arp, Mila Jhamai, Marijn Verkerk, Lizbeth Herrera and Marjolein Peters, MSc, and Carolina Medina-Gomez, MSc, for their help in creating the GWAS database, and Karol Estrada, PhD, Yurii Aulchenko, PhD, and Carolina Medina-Gomez, MSc, for the creation and analysis of imputed data. We would like to thank Karol Estrada PhD, Fernando Rivadeneira PhD, Tobias A. Knoch PhD, Anis Abuseiris and Rob de Graaf (Erasmus MC Rotterdam,

The Netherlands), for their help in creating GRIMP, and we thank BigGRID, MediGRID, and Services@MediGRID/D-Grid, (funded by the German Bundesministerium fuer Forschung und Technology; grants 01 AK 803 A-H, 01 IG 07015 G) for access to their grid computing resources.

SardiNIA would like to thank all the volunteers who generously participated in this study, Monsignore Piseddu, Bishop of Ogliastra and the mayors and citizens of the Sardinian towns (Lanusei, Ilbono, Arzana, and Elini). This work was supported by the Intramural Research Program of the National Institute on Aging (NIA), National Institutes of Health (NIH). The SardiNIA ("Progenia") team was supported by Contract NO1-AG-1–2109 from the NIA; the efforts of GRA were supported in part by contract 263-MA-410953 from the NIA to the University of Michigan and by research grant HG002651 and HL084729 from the NIH (to GRA).

SHIP is part of the Community Medicine Research net of the University of Greifswald, Germany, which is funded by the Federal Ministry of Education and Research (grants no. 01ZZ9603, 01ZZ0103, and 01ZZ0403), the Ministry of Cultural Affairs as well as the Social Ministry of the Federal State of Mecklenburg-West Pomerania, and the network 'Greifswald Approach to Individualized Medicine (GANI_MED)' funded by the Federal Ministry of Education and Research (grant 03IS2061A). Genome-wide data have been supported by the Federal Ministry of Education and Research (grant no. 03ZIK012) and a joint grant from Siemens Healthcare, Erlangen, Germany and the Federal State of Mecklenburg- West Pomerania. The University of

Greifswald is a member of the Caché Campus program of the InterSystems GmbH.

Sorbs , this work was supported by grants from the German Research Council (SFB- 1052 ""Obesity mechanisms"" to Michael Stumvoll, Anke Tönjes and Peter Kovacs), from the German Diabetes Association (to Anke Tönjes and Peter Kovacs) and from the DHFD (Diabetes Hilfs- und Forschungsfonds Deutschland to Michael Stumvoll and Peter Kovacs). Peter Kovacs is funded by the Boehringer Ingelheim Foundation. We thank all those who participated in the study. Sincere thanks are given to Knut Krohn (Microarray Core Facility of the Interdisciplinary Centre for Clinical Research, University of Leipzig) for the genotyping support. Reedik Mägi is funded by European Commission under the Marie Curie Intra-European Fellowship and by Estonian Government (grant #SF0180142s08)."

TwinsUK, the study was funded by the Wellcome Trust; European Community's Seventh Framework Programme (FP7/2007-2013). The study also receives support from the National Institute for Health Research (NIHR)- funded BioResource, Clinical Research Facility and Biomedical Research Centre based at Guy's and St Thomas' NHS Foundation Trust in partnership with King's College London. SNP Genotyping was performed by The Wellcome Trust Sanger Institute and National Eye Institute via NIH/CIDR

WGHS, the WGHS is supported by HL043851 and HL080467 from the National Heart, Lung, and Blood Institute and CA047988 from the National Cancer Institute with collaborative scientific support and funding for genotyping provided by Amgen.

T2D-WTCCC , the genotyping and analysis was funded in part through Andrew Morris funding from the Wellcome Trust (grant numbers 081682, 098017 and 090532); Mark McCarthy funding from the Wellcome Trust (grant numbers 090532, 085301, 081917, 098381, 090367 and 083270), European Commission (HEALTH-F4-2007-201413) and MRC G0601261. C.M. Lindgren is a Wellcome Trust Research Career Development Fellow (086596/Z/08/Z). M. McCarthy is an NIHR Senior Investigator and a Wellcome Trust Senior Investigator. R. Mägi is funded by European Commission under the Marie Curie Intra-European Fellowship and by Estonian Government (grant #SF0180142s08). Andrew Morris is a Wellcome Trust Senior Fellow in Basic Biomedical Science (WT098017).

YFS, The Young Finns Study has been financially supported by the Academy of Finland: grants 134309 (Eye), 126925, 121584, 124282, 129378 (Salve), 117787 (Gendi), and 41071 (Skidi), the Social Insurance Institution of Finland, Kuopio, Tampere and Turku University Hospital Medical Funds (grant 9M048 and 9N035 for TeLeht), Juho Vainio Foundation, Paavo Nurmi Foundation, Finnish Foundation of Cardiovascular Research and Finnish Cultural Foundation, Tampere Tuberculosis Foundation and Emil Aaltonen Foundation (T.L). The THL DNA laboratory for its skillful work to produce the DNA samples used in this study and the expert technical assistance in the statistical analyses by Ville Aalto and Irina Lisinen are gratefully acknowledged.

2nd Stage studies (Metabochip)

DIAGEN, the DIAGEN study was supported by the Commission of the European Communities, Directorate C - Public Health and Risk Assessment, Health & Consumer Protection, Grant Agreement number - 2004310 and by the Dresden University of Technology Funding Grant, Med Drive. We are grateful to all of the patients who cooperated in this study and to their referring physicians and diabetologists in Saxony.

DPS.DRSEXTRA.FIN-D2D2007.FUSIONS2.METSIM (DPS), the DPS has been financially supported by grants from the Academy of Finland (117844 and 40758, 211497, and 118590; The EVO funding of the Kuopio University Hospital from Ministry of Health and Social Affairs (5254), Finnish Funding Agency for Technology and Innovation (40058/07), Nordic Centre of Excellence on Systems biology in controlled dietary interventions and cohort studies, SYSDIET (070014), The Finnish Diabetes Research Foundation, Yrjö Jahnsson Foundation (56358), Sigrid Juselius Foundation, Juho Vainio Foundation and TEKES grants 70103/06 and 40058/07.

DPS.DRSEXTRA.FIN-D2D2007.FUSIONS2.METSIM (DR'S EXTRA), the DR's EXTRA Study was supported by the Ministry of Education and Culture of Finland (627;2004-2011), Academy of Finland (102318; 123885), Kuopio University Hospital, Finnish Diabetes Association, Finnish Foundations for Cardiovascular Research, Päivikki and Sakari Sohlberg Foundation, by European Commission FP6 Integrated Project (EXGENESIS); LSHM-CT-2004-005272, City of Kuopio and Social Insurance Institution of Finland (4/26/2010).

DPS.DRSEXTRA.FIN-D2D2007.FUSIONS2.METSIM (FIN-D2D 2007), the FIN-D2D study has been financially supported by the hospital districts of Pirkanmaa,

South Ostrobothnia, and Central Finland, the Finnish National Public Health Institute (current National Institute for Health and Welfare), the Finnish Diabetes Association, the Ministry of Social Affairs and Health in Finland, the Academy of Finland (grant number 129293),Commission of the European Communities, Directorate C-Public Health (grant agreement no. 2004310) and Finland’s Slottery Machine Association.

DPS.DRSEXTRA.FIN-D2D2007.FUSIONS2.METSIM (FUSION Stage 2 cases and controls), support for FUSION was provided by NIH grants R01-DK062370 (to M.B.), R01-DK072193 (to K.L.M.), and intramural project number 1Z01-HG000024 (to F.S.C.). Genome-wide genotyping was conducted by the Johns Hopkins University Genetic Resources Core Facility SNP Center at the Center for Inherited Disease Research (CIDR), with support from CIDR NIH contract no. N01-HG-65403.

DPS.DRSEXTRA.FIN-D2D2007.FUSIONS2.METSIM (METSIM), the METSIM study was funded by the Academy of Finland (grants no. 77299 and 124243).
Ely Ely We are grateful to all the volunteers and to the staff of St. Mary's Street Surgery, Ely and the study team. The Ely Study was funded by the MRC (MC_U106179471) and Diabetes UK. Genotyping in the Ely and Fenland studies was supported in part by an MRC-GlaxoSmithKline pilot programme grant (G0701863).

EPIC, pleases see above.

Fenland, please see above.

Health 2006, the Health2006 study was financially supported by grants from the Velux Foundation; the Danish Medical Research Council, Danish Agency for Science, Technology and Innovation; the Aase and Ejner Danielsens Foundation; ALK-Abello´ A/S (Hørsholm, Denmark), Timber Merchant Vilhelm Bangs Foundation, MEKOS Laboratories Denmark) and Research Centre for Prevention and Health, the Capital Region of Denmark. This project was also funded by the Lundbeck Foundation and produced by The Lundbeck Foundation Centre for Applied Medical Genomics in Personalised Disease Prediction, Prevention and Care (LuCamp,www.lucamp.org). The Novo Nordisk Foundation Center for Basic Metabolic Research is an independent Research Center at the University of Copenhagen partially funded by an unrestricted donation from the Novo Nordisk Foundation (www.metabol.ku.dk).

HUNT/TROMSO (HUNT2), the Nord-Trøndelag Health Study (The HUNT Study) is a collaboration between HUNT Research Centre (Faculty of Medicine, Norwegian University of Science and Technology NTNU), Nord-Trøndelag County Council, Central Norway Health Authority, and the Norwegian Institute of Public Health.

HUNT/TROMSO (Tromsø), University of Tromsø, Norwegian Research Council (project number 185764)

Inter99 (INTER99), The Inter99 was initiated by Torben Jørgensen (PI), Knut Borch-Johnsen (co-PI), Hans Ibsen and Troels F. Thomsen. The steering committee comprises the former two and Charlotta Pisinger. The study was financially supported by research grants from the Danish Research Council, the

Danish Centre for Health Technology Assessment, Novo Nordisk Inc., Research Foundation of Copenhagen County, Ministry of Internal Affairs and Health, the Danish Heart Foundation, the Danish Pharmaceutical Association, the Augustinus Foundation, the Ib Henriksen Foundation, the Becket Foundation, and the Danish Diabetes Association. This project was also funded by the Lundbeck Foundation and produced by The Lundbeck Foundation Centre for Applied Medical Genomics in Personalised Disease Prediction, Prevention and Care (LuCamp,www.lucamp.org). The Novo Nordisk Foundation Center for Basic Metabolic Research is an independent Research Center at the University of Copenhagen partially funded by an unrestricted donation from the Novo Nordisk Foundation (www.metabol.ku.dk).

KORA F3 and KORA S4, please see above.

NSHD , this work was funded by the Medical Research Council (MC_UU_12019/1), the British Heart Foundation (RG/10/12/28456) and the Wellcome Trust (088869/B/09/Z). We are very grateful to the members of this birth cohort for their continuing interest and participation in the study. We would like to acknowledge the Swallow group, UCL, who performed the DNA extractions (Rousseau, et al 2006). DOI: 10.1111/j.1469-1809.2006.00250.x

THISEAS THISEAS Recruitment for THISEAS was partially funded by a research grant (PENED 2003) from the Greek General Secretary of Research and Technology; we thank all the dieticians and clinicians for their contribution to the project. Genotyping was funded by the Wellcome Trust (core grant 098051). We like to thank the members of the Wellcome Trust Sanger Institute Genotyping Facility.

CM.GOYA (MALE GOYA), this study was conducted as part of the activities of the 'Gene-diet Interactions in Obesity' project (GENDINOB, www.gendinob.dk) and the MRC centre for Causal Analyses in Translational Epidemiology (MRC CAiTE). We thank the staff of the Copenhagen City Heart Study for their skillful examination of the study subjects in collection of baseline and follow-up data.

HELIC-HA, this work was funded by the Wellcome Trust (098051) and the European Research Council (ERC-2011-StG 280559-SEPI). The MANOLIS cohort is named in honour of Manolis Giannakakis, 1978-2010. We thank the residents of Anogia and surrounding Mylopotamos villages, and of the Pomak villages, for taking part. The HELIC study has been supported by many individuals who have contributed to sample collection (including Antonis Athanasiadis, Olina Balafouti, Christina Batzaki, Georgios Daskalakis, Eleni Emmanouil, Chrisoula Giannakaki, Margarita Giannakopoulou, Anastasia Kaparou, Vasiliki Kariakli, Stella Koinaki, Dimitra Kokori, Maria Konidari, Hara Koundouraki, Dimitris Koutoukidis, Vasiliki Mamakou, Eirini Mamalaki, Eirini Mpamiaki, Maria Tsoukana, Dimitra Tzakou, Katerina Vosdogianni, Niovi Xenaki, Eleni Zengini), data entry (Thanos Antonos, Dimitra Papagrigoriou, Betty Spiliopoulou), sample logistics (Sarah Edkins, Emma Gray), genotyping (Robert Andrews, Hannah Blackburn, Doug Simpkin, Siobhan Whitehead), research administration (Anja Kolb-Kokocinski, Carol Smee, Danielle Walker) and informatics (Martin Pollard, Josh Randall).

HELIC-HP, this work was funded by the Wellcome Trust (098051) and the European Research Council (ERC-2011-StG 280559-SEPI). The MANOLIS cohort is named in honour of Manolis Giannakakis, 1978-2010. We thank the residents of Anogia and surrounding Mylopotamos villages, and of the Pomak

villages, for taking part. The HELIC study has been supported by many individuals who have contributed to sample collection (including Antonis Athanasiadis, Olina Balafouti, Christina Batzaki, Georgios Daskalakis, Eleni Emmanouil, Chrisoula Giannakaki, Margarita Giannakopoulou, Anastasia Kaparou, Vasiliki Kariakli, Stella Koinaki, Dimitra Kokori, Maria Konidari, Hara Koundouraki, Dimitris Koutoukidis, Vasiliki Mamakou, Eirini Mamalaki, Eirini Mpamiaki, Maria Tsoukana, Dimitra Tzakou, Katerina Vosdogianni, Niovi Xenaki, Eleni Zengini), data entry (Thanos Antonos, Dimitra Papagrigoriou, Betty Spiliopoulou), sample logistics (Sarah Edkins, Emma Gray), genotyping (Robert Andrews, Hannah Blackburn, Doug Simpkin, Siobhan Whitehead), research administration (Anja Kolb-Kokocinski, Carol Smee, Danielle Walker) and informatics (Martin Pollard, Josh Randall).

HERITAGE, the HERITAGE Family Study was supported for 15 years by multiple grants from the National Heart, Lung, and Blood Institute to C. Bouchard, A.S. Leon, D.C. Rao, J.S. Skinner and J.H. Wilmore. The current work was supported in part by grant HL-45670 to C. Bouchard, T. Rankinen and D.C. Rao.

LOLIPOP_EW_A + LOLIPOP_EW_P + LOLIPOP_EW610, the LOLIPOP study is supported by the National Institute for Health Research (NIHR) Comprehensive Biomedical Research Centre Imperial College Healthcare NHS Trust, the British Heart Foundation (SP/04/002), the Medical Research Council (G0601966,G0700931), the Wellcome Trust (084723/Z/08/Z) the NIHR (RP-PG-0407-10371),European Union FP7 (EpiMigrant, 279143) and Action on Hearing Loss (G51). The work was carried out in part at the NIHR/Wellcome Trust Imperial Clinical Research Facility. The views expressed are those of the author(s) and not necessarily those of the Imperial College Healthcare NHS

Trust, the NIHR or the Department of Health. We thank the participants and research staff who made the study possible.

NTRNESDA, please see above.

QFS, the Quebec Family Study (QFS) was funded by multiple grants from the Medical Research Council of Canada and the Canadian Institutes for Health Research. This work was supported by a team grant from the Canadian Institutes for Health Reseaerch (FRCN-CCT-83028)

TRAILS-Pop (TRAILS), this research is part of the TRacking Adolescents' Individual Lives Survey (TRAILS). Participating centers of TRAILS include various departments of the University Medical Center and University of Groningen, the Erasmus University Medical Center Rotterdam, the University of Utrecht, the Radboud Medical Center Nijmegen, and the Parnassia Bavo group, all in the Netherlands. TRAILS has been financially supported by various grants from the Netherlands Organization for Scientific Research NWO (Medical Research Council program grant GB-MW 940-38-011; ZonMW Brainpower grant 100-001-004; ZonMw Risk Behavior and Dependence grants 60-60600-97-118; ZonMw Culture and Health grant 261-98-710; Social Sciences Council medium-sized investment grants GB-MaGW 480-01-006 and GB-MaGW 480-07-001; Social Sciences Council project grants GB-MaGW 452-04-314 and GB-MaGW 452-06-004; NWO large-sized investment grant 175.010.2003.005; NWO Longitudinal Survey and Panel Funding 481-08-013), the Dutch Ministry of Justice (WODC), the European Science Foundation (EuroSTRESS project FP-006), Biobanking and Biomolecular Resources Research Infrastructure BBMRI-NL (CP 32), and the participating universities. We are grateful to all

adolescents, their parents and teachers who participated in this research and to everyone who worked on this project and made it possible. Statistical analyses were carried out on the Genetic Cluster Computer (http://www.geneticcluster.org), which is financially supported by the Netherlands Scientific Organization (NWO 480-05-003) along with a supplement from the Dutch Brain Foundation.
